# MnO_2_ Nanosponge‐Accelerated Cas12a *Trans*‐Cleavage: Breaking the Kinetic Barrier for In Vivo RNA Imaging

**DOI:** 10.1002/advs.202511942

**Published:** 2025-10-14

**Authors:** Wen‐jing Liu, Lu‐yao Wang, Fei Ma, Chun‐yang Zhang

**Affiliations:** ^1^ School of Chemistry and Chemical Engineering State Key Laboratory of Digital Medical Engineering Southeast University Nanjing 211189 China

**Keywords:** cancer diagnosis, CRISPR/Cas12a, fluorescent detection, RNA imaging, signal amplification

## Abstract

CRISPR/Cas12a system has emerged as a promising tool for in vitro biosensing, but its in vivo applications are hindered by its inefficient intracellular delivery and suboptimal *trans*‐cleavage kinetics. To address these challenges, a Cas12a@MnO_2_ nanosponge (hMNS) nanoprobe is constructed, in which hMNS as both a degradable carrier and an accelerator of CRISPR/Cas12a system for efficient imaging of RNA in living cells. The Cas12a@hMNS nanoprobe is obtained via a one‐step co‐assembly process. It not only facilitates synchronous cellular uptake and glutathione (GSH)‐responsive release of CRISPR/Cas12a components, but also supplies adequate Mn^2+^ cofactors to improve the *trans*‐cleavage activity of Cas12a. This dual‐function probe can break the kinetic barrier of conventional CRISPR/Cas12a systems due to its unique characteristics of effective cellular internalization, rapid intracellular release, and accelerated signal gain, enabling sensitive detection of mRNA down to 63.6 pM without pre‐amplification. Moreover, the Cas12a@hMNS nanoprobe can profile endogenous mRNA at the single‐cell level, discriminate breast cancer tissues from healthy counterparts, and real‐time visualize mRNA dynamics in living cells with exceptional spatiotemporal precision. Importantly, the elongation‐blocked (EB) activator‐modulated CRISPR/Cas12a system can be extended to detect various intracellular biomarkers, holding promising applications in clinical diagnosis, treatment, and surveillance.

## Graphical abstract

A Cas12a@MnO_2_ nanosponge (hMNS) nanoprobe is engineered to overcome the intracellular delivery and kinetic barrier of the conventional CRISPR/Cas12a system via GSH‐responsive release and Mn^2^⁺‐enhanced *trans*‐cleavage. It enables preamplification‐free detection of mRNA with single‐cell sensitivity, differentiation of mRNA expression between breast cancer tissues and their healthy counterparts, and real‐time tracking of mRNA dynamics in living cells.

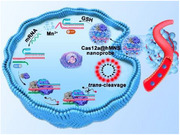

## 1 Introduction

CRISPR (Clustered regularly interspaced short palindromic repeats) constitutes an adaptive prokaryotic immune system that provides sequence‐specific defense against foreign genetic elements such as bacteriophages and conjugative plasmids.^[^ ^1^, ^2^ ^]^ CRISPR‐associated (Cas) proteins allow sequence‐specific recognition and catalytic cleavage of nucleic acids, which have greatly broadened our toolkits for precision genome editing,^[^ ^3^, ^4^ ^]^ transcriptional regulation,^[^ ^5^ ^]^ and molecular diagnostics.^[^ ^6^, ^7^, ^8^, ^9^ ^]^ As a representative member of the CRISPR/Cas families, Cas12a (formerly Cpf1) is a class 2 type V CRISPR‐associated nuclease that binds with a crRNA.^[^ ^10^, ^11^ ^]^ The crRNA contains a hairpin “scaffold” motif for binding with Cas12a and a programmable “spacer” motif for recognizing the protospacer of target DNA (activator).^[^ ^12^ ^]^ Different from other CRISPR/Cas families, CRISPR/Cas12a can not only specifically cleave dsDNA (*cis*‐cleavage) but also non‐specifically cleave ssDNA (*trans*‐cleavage).^[^ ^13^, ^14^, ^15^, ^16^, ^17^ ^]^ These unique catalytic properties have been harnessed to engineer Cas12‐based nucleic acid detection strategies (e.g., DETECTR,^[^ ^14^ ^]^ HOLMES,^[^ ^18^ ^]^ and CONAN^[^ ^19^ ^]^), greatly increasing the versatility and flexibility of Cas12‐based biosensor design.^[^ ^8^, ^20^, ^21^, ^22^, ^23^ ^]^ Notably, the *cis*‐cleavage activity of Cas12a is only 1 turnover, while its *trans*‐cleavage exhibits rapid initial kinetics (≈1250 s^−1^) and then decasy to ≈17 s^−1^,^[^ ^14^ ^]^ posing a challenge for live‐cell applications. Although the low activity of Cas12a can be circumvented by integrating diverse nucleic acid amplification strategies in biosensing applications,^[^ ^21^, ^24^, ^25^, ^26^, ^27^ ^]^ their implementation in intracellular bioimaging faces several barriers: 1) the practical impossibility of controlled amplification in cells, 2) significant time delays in signal gain, and 3) unavoidable perturbation of native cellular physiology and molecular profiles. As a result, it is urgently desired to develop a simple, rapid, transfectant‐free method to enhance the sensing performance of CRISPR/Cas12a in living cells.

Previous studies have demonstrated that Mn^2+^ not only replaces Mg^2+^ to coordinate in the RuvC endonuclease domains of Cas12a,^[^ ^28^, ^29^, ^30^ ^]^ but also serves as the accelerator to enhance the cleavage activity of Cas12a.^[^ ^31^, ^32^ ^]^ Taking into account the fact that the Cas12a‐based sensing system involves multiple components (e.g., Cas12a protein, crRNA, DNA activator, and fluorescent reporter probe), effective and synchronized co‐delivery of these components into living cells is a prerequisite for the CRISPR/Cas12a‐based intracellular imaging. Although lipid‐, polymer‐, and DNA nanoclew‐based carriers have achieved efficient delivery of CRISPR,^[^ ^33^, ^34^, ^35^ ^]^ they suffer from inefficient co‐encapsulation of multicomponent, dependence on exogenous cofactors, nonspecific endosomal release, and off‐target cleavage. As an excellent three‐dimensional (3D) nanomaterial, honeycomb MnO_2_ nanosponge (hMNS) has attracted great interest in biomedical applications due to its facile preparation, large surface area, low cytotoxicity, and good biocompatibility.^[^ ^36^, ^37^ ^]^ The hMNS can function as a carrier for delivering various payloads (e.g., nucleic acids,^[^ ^36^, ^37^, ^38^, ^39^ ^]^ proteins,^[^ ^40^ ^]^ and drugs^[^ ^41^ ^]^) into cells. After being endocytosed into cells, hMNS can be degraded by endogenous reductants (e.g., glutathione (GSH) that is overexpressed in tumor cells ^[^ ^42^, ^43^, ^44^, ^45^ ^]^), leading to the rapid liberation of payloads. In this research, we construct a Cas12a@MnO_2_ nanosponge (hMNS) nanoprobe with hMNS as both a carrier and an accelerator of CRISPR/Cas12a system to break the kinetic barrier for efficient in vivo imaging of RNA. The Cas12a@hMNS nanoprobe is obtained by simply assembling all the components of CRISPR/Cas12a sensing system on the hMNS nanocarrier surface. Compared with existing Cas12a enhancement strategies (e.g., allosteric crRNA engineering^[^ ^12^ ^]^ and framework‐assisted stabilization^[^ ^15^ ^]^), our hMNS nanocarrier has three critical advantages: 1) elimination of complex oligonucleotide engineering through intrinsic Mn^2+^ release, 2) facilitating the translocation of CRISPR/Cas12a‐based sensing system into living cells, and 3) supplying sufficient Mn^2+^ to improve the *trans*‐cleavage activity of Cas12a. This self‐sustained Cas12a@hMNS nanoprobe demonstrates superior performance compared with commercial carriers, and it can achieve high signal amplification gain without detectable leakage, facilitating sensitive RNA sensing across in vitro and in vivo.

## 2 Results and Discussion

### 2.1 Design Principle of mRNA Imaging in Live Cells

**Scheme 1:**
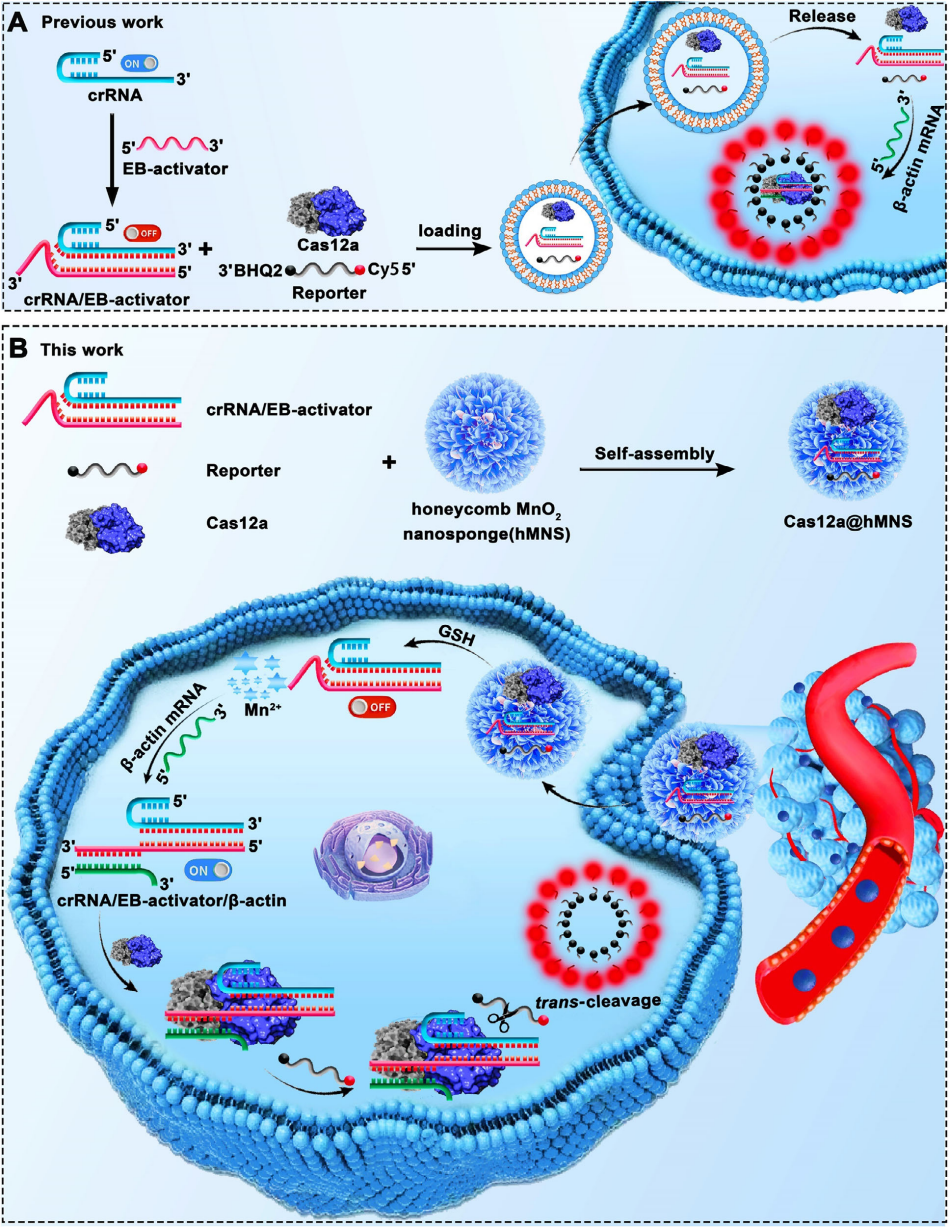
A) Schematic showing the conventional design of CRISPR/Cas12a‐based sensing system. B) Design principle of a Cas12a@hMNS nanoprobe with the integration of hMNS and CRISPR/Cas12a‐based sensing system for live‐cell mRNA imaging.

As illustrated in **Scheme** **1**, we construct a Cas12a@hMNS nanoprobe that integrates a honeycomb MnO_2_ nanosponge (hMNS) with a CRISPR/Cas12a‐based sensing system for efficient imaging of mRNA in living cells. β‐actin mRNA was chosen as a model analyte for proof‐of‐concept. To achieve target mRNA‐specific response, we appended a short extension fragment to the 3′‐end of the ssDNA activator (elongation‐blocked activator, EB‐activator) and engineered it to block the crRNA spacer/repeat junction, making the whole CRISPR/Cas12a system in an “OFF” state. The hMNS nanocarrier is responsive to endogenous GSH and simultaneously serves as a precursor of the CRISPR/Cas12a accelerator, facilitating the imaging of intracellular mRNA. Upon the delivery of the CRISPR/Cas12a‐based sensing system into living cells, the hMNS nanocarrier is disassembled by endogenous GSH, resulting in the generation of Mn^2+^ and the programmed release of CRISPR/Cas12a system components (i.e., Cas12a, crRNA/EB‐activator, and reporter). Subsequently, the endogenous mRNA hybridizes with the 3′ toehold domain in the EB‐activator of the crRNA/EB‐activator via toehold‐mediated strand displacement to form an overhanging mRNA/EB‐activator heteroduplex, accompanied by the activation of Cas12a/crRNA. The activated Cas12a/crRNA is switched from “OFF” to “ON” state, inducing Mn^2+^‐accelerated *trans*‐cleavage of Cy5/BHQ2 dual‐labeled reporter and restoring the Cy5 fluorescence signal (Figure S1, Supporting Information). Notably, both the hMNS‐programmed delivery of CRISPR/Cas12a‐based sensing systems and Mn^2+^‐enhanced *trans*‐cleavage activity of Cas12a facilitate real‐time imaging of mRNA in living cells.

### 2.2 Assessing the Inhibitory Effect of Various EB‐activators on the Trans‐Cleavage Activity of Cas12a

**Figure 1:**
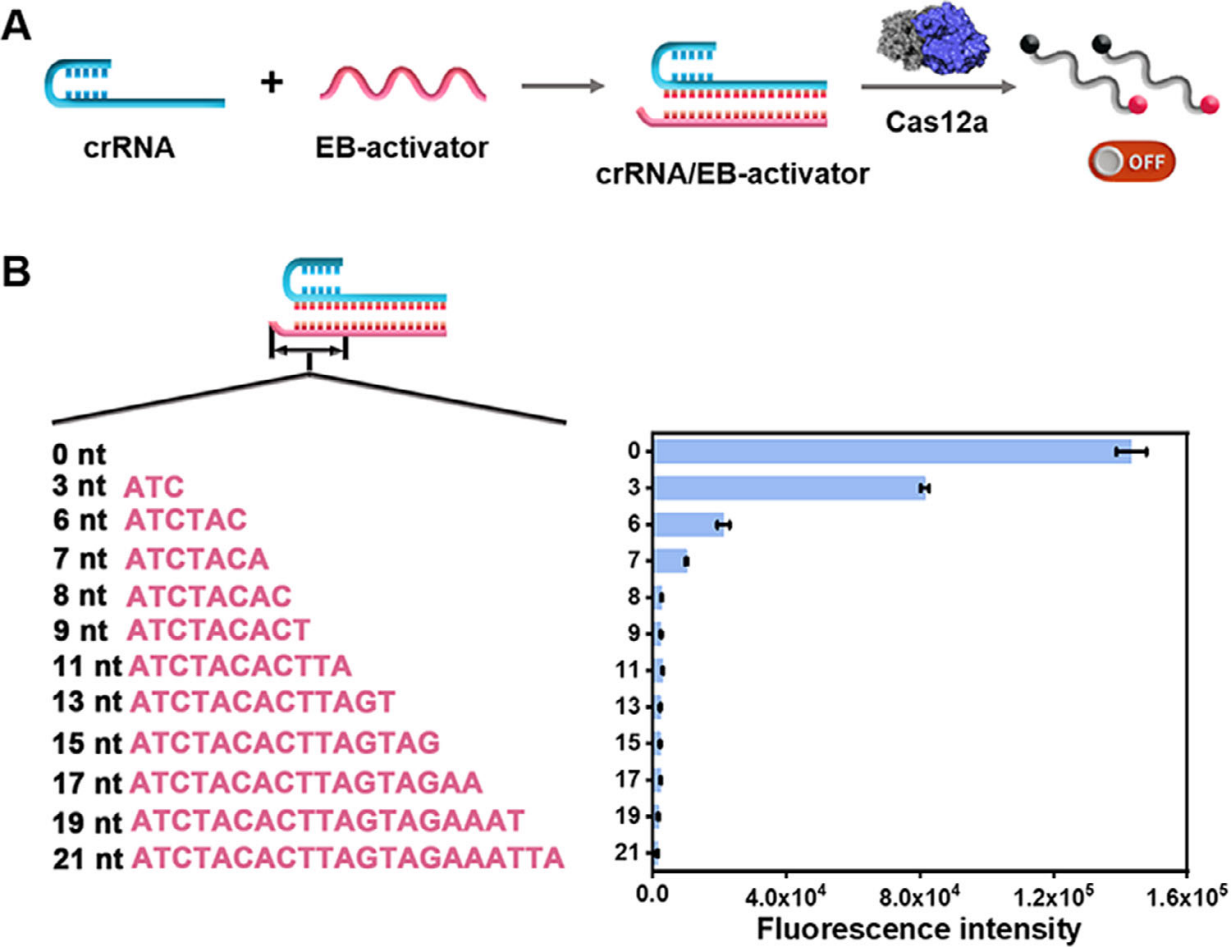
A) Schematic of the pre‐hybridization of EB‐activator with crRNA to prevent the assembly of crRNA with Cas12a. B) Sequences of EB‐activator with various extension lengths (left panel) and their inhibitory effect on the *trans*‐cleavage activity of Cas12a (right panel). Data are presented as mean ± SD (*n* = 3).

In the CRISPR/Cas12a system, the binding of Cas12a to the handle‐like crRNA repeat region is essential for the activator recognition by the crRNA spacer region.^[^ ^10^, ^46^ ^]^ Thus, the blocking of crRNA repeat/spacer junction may effectively inhibit the *trans*‐cleavage activity of Cas12a, inducing a long‐term low background. In this assay, we engineered a series of elongation‐blocked (EB)‐activators with various lengths (i.e., EB‐3, EB‐6, EB‐7, EB‐8, EB‐9, EB‐11, EB‐13, EB‐15, EB‐17, EB‐19, and EB‐21, where the numbers represent the length of the 3′‐extension fragment paired with the repeat region) (Figure 1B). EB‐activator can hybridize with crRNA to form the crRNA/EB‐activator duplex, blocking the conformation changes of crRNA and hindering the interaction of Cas12a with crRNA (Figure 1A). In theory, the EB‐activator with too short a length cannot effectively block the Cas12a activity, but excessively long EB‐activator might impede the complete activation of Cas12a. The increase of 3′‐elongated fragment length induces the decrease of Cy5 fluorescence intensity, with a plateau being achieved at EB‐8 (Figure 1B). This result suggests that sufficient complementary bases between the EB‐activator and crRNA repeat region are required to induce efficient inhibition of Cas12a *trans*‐cleavage activity, and that the attachment of more than 8 nucleotides at the 3′‐end of the activator can completely inhibit the *trans*‐cleavage activity of Cas12a.

### 2.3 Characterization and Validation of Cas12a@hMNS Nanoprobe

**Figure 2:**
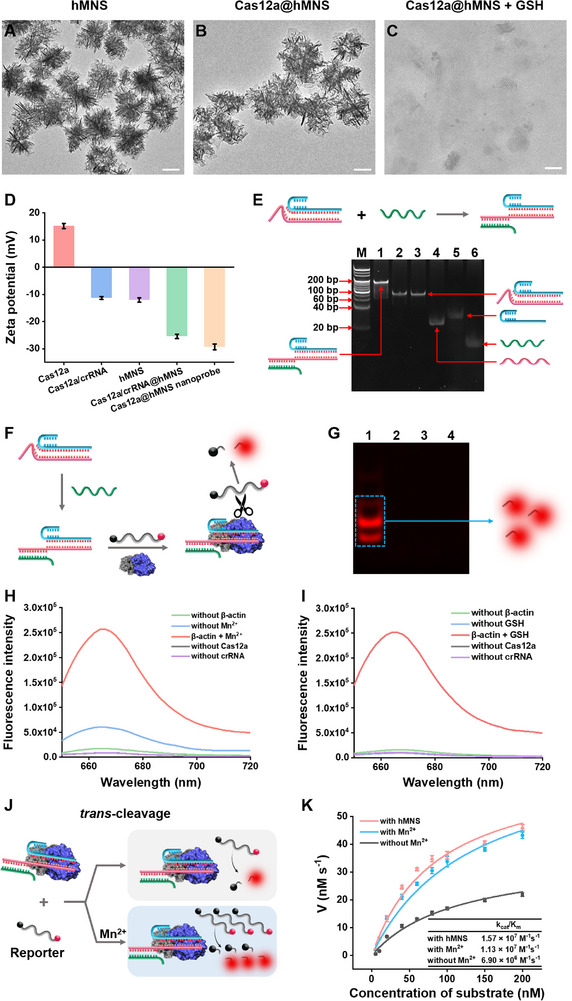
A–C) TEM images of hMNS (A), Cas12a@hMNS nanoprobe (B), and Cas12a@hMNS nanoprobe + GSH (C). Scale bar: 100 nm. D) Zeta potential of Cas12a Cas12a/crRNA, hMNS, Cas12a/crRNA@hMNS, and Cas12a@hMNS nanoprobe. E) 14% nondenaturing PAGE analysis of β‐actin mRNA‐induced crRNA activation. Lane 1, crRNA/EB‐activator + β‐actin mRNA; lane 2, crRNA/EB‐activator; lane 3, crRNA/EB‐activator; lane 4, EB‐activator; lane 5, crRNA; lane 6, β‐actin mRNA; lane M, DNA marker; F) Schematic showing Cas12a/crRNA‐mediated *trans*‐cleavage of reporters. G) 14% nondenaturing PAGE analysis of the activated Cas12a/crRNA‐mediated cleavage products. Lane 1, crRNA/EB‐activator + Cas13a + reporter + β‐actin mRNA; lane 2, crRNA/EB‐activator + Cas13a + reporter; lane 3, crRNA/EB‐activator + reporter + β‐actin mRNA; lane 4, the synthetic reporter. H) Fluorescence spectra of free CRISPR/Cas12a‐based sensing system under different reaction conditions. I) Fluorescence spectra of Cas12a@hMNS nanoprobe under different reaction conditions. J) Schematic of Cas12a *trans*‐cleavage with (bottom) and without (top) Mn^2+^. K) The *trans*‐cleavage kinetic profiles of Cas12a under different treatment conditions. Data are presented as mean ± SD (*n* = 3).

The Cas12a@hMNS nanoprobe is facilely synthesized by one‐step mixing hMNS with the components of the CRISPR/Cas12a‐based sensing system, and subsequently characterized by transmission electron microscopy (TEM) and ζ‐potential analysis. The hMNS exhibits a sponge‐like morphology with good dispersity (**Figure** **2**A) and a zeta potential of ‐12.07 mV (Figure 2D, purple column). Due to the positive charge of Cas12a protein (15.13 mV, Figure 2D, red column) and the nanoassembly obtained by hMNS and oligonucleotides via electrostatic adsorption, the components of the CRISPR/Cas12a‐based sensing system can be further assembled to form a Cas12a/reporter/crRNA/EB‐activator@hMNS nanoprobe (namely the Cas12a@hMNS nanoprobe). The Cas12a@hMNS nanoprobe shows a sponge‐like structure (Figure 2B) with a zeta potential of −29.3 mV (Figure 2D, orange column). Notably, the Cas12a@hMNS nanoprobe can rapidly degrade in an intracellular reductive environment with high GSH level (Figure 2C; Figure S2, Supporting Information). These results demonstrate the successful construction of a Cas12a@hMNS nanoprobe.

We used 14% nondenaturing PAGE to analyze β‐actin mRNA‐induced activation of crRNA. As displayed in Figure 2E, the hybridization of crRNA with EB‐activator forms a crRNA/EB‐activator heteroduplex (Figure 2E, lane 3). When β‐actin mRNA is absent, an intact band of crRNA/EB‐activator is visualized (Figure 2E, lane 2), which has the same mobility as the original crRNA/EB‐activator (Figure 2E, lane 3). Upon the addition of β‐actin mRNA, the band of the crRNA/EB‐activator/β‐actin mRNA ternary complex appears (Figure 2E, lane 1), indicating the successful hybridization of β‐actin mRNA with the 3′ toehold domain of the EB‐activator. Subsequently, we used 14% nondenaturing PAGE to analyze the Cas12a/crRNA‐mediated *trans*‐cleavage products with direct excitation of Cy5 (Figure 2F,G). In the absence of β‐actin mRNA, the reporter remains intact, and no Cy5 band is observed (Figure 2G, lane 2). In contrast, the presence of β‐actin mRNA induces a distinct Cy5 band (Figure 2G, lane 1), suggesting that β‐actin mRNA can activate the Cas12a/crRNA to effectively cleave reporters. In contrast, when Cas12a (Figure 2G, lane 3) is absent, no band of Cy5 appears, indicating the critical role of Cas12a protein in the CRISPR/Cas12a system.

We conducted fluorescence measurements to verify the feasibility of this Cas12a@hMNS nanoprobe (Figure 2H). When β‐actin mRNA is absent, the CRISPR/Cas12a system remains “OFF” state with a negligible fluorescence response (Figure 2H, green curve). In contrast, a high fluorescence response is detected when β‐actin mRNA is present (Figure 2H, blue curve), and the addition of Mn^2+^ induces a further increase in fluorescence response (Figure 2H, red curve), indicating the significant improvement of Cas12a *trans‐*cleavage activity induced by Mn^2+^. Moreover, only a pretty weak fluorescence response is observed when the whole reaction is performed without either Cas12a (Figure 2H, grey curve) or crRNA (Figure 2H, purple curve), confirming that these components are necessary for live‐cell imaging. In contrast to the free CRISPR/Cas12a system, this Cas12a@hMNS nanoprobe remains inert toward β‐actin mRNA without the involvement of GSH, which shows no difference from the control groups (Figure 2I, blue curve). The passivation of CRISPR/Cas12a facilitates efficient and safe delivery of the CRISPR/Cas12a‐based sensing system into living cells. Notably, the addition of GSH induces a significantly enhanced fluorescence response (Figure 2I, red curve) that is comparable to that generated by the Mn^2+^‐accelerated CRISPR/Cas12a system (Figure 2H, red curve), suggesting that hMNS is not only an efficient CRISPR/Cas12a system delivery carrier, but also a Mn^2+^ donor that greatly enhances the *trans*‐cleavage of Cas12a.

The *trans*‐cleavage kinetics of Cas12a with and without hMNS (Figure 2J) were further evaluated by measuring the initial velocity (*V*) in response to varying concentrations of reporters from 0 to 200 nM in 10 min reaction at 37 °C (Figure S3, Supporting Information). The Michaels‐Menton constant (*K*_m_) is calculated based on Equation (1).

(1) $$V=V_{max}\left[S\right]/\left(K_{m}+\left[S\right]\right)$$

where *V*_max_, *K*_m_, and [*S*] are the maximum initial velocity, the Michaelis‐Menten constant, and the concentration of reporter, respectively. The catalytic efficiency (*k*_cat_/*K*_m_) is obtained according to Equation (2).

(2) $$k_{cat}=V_{max}/E_{0}$$

where *E*_0_ is the Cas12a concentration. As depicted in Figure 2K, in the absence of Mn^2+^ (Figure 2K, grey curve), the turnover number (*k*_cat_) is measured to be 0.79 s^−1^, and the catalytic efficiency (*k*_cat_/*K*_m_) is calculated to be 6.90 × 10^6^ M^−1^ s^−1^. Upon the addition of hMNS (Figure 2K, red curve), the *k*_cat_ value is enhanced to 1.37 s^−1^, and the *k*_cat_/*K*_m_ value is improved to 1.57 × 10^7^ M^−1^ s^−1^, which is consistent with the *trans*‐cleavage kinetics of Cas12a in response to exogenous Mn^2^⁺ (Figure 2K, blue curve, *k*_cat_/*K*_m_ = 1.13 × 10^7^ M^−1^ s^−1^), suggesting that Mn^2+^ can effectively improve the *trans*‐cleavage activity of Cas12a. The improved *trans*‐cleavage activity may be ascribed to the fact that Mn^2+^ has a higher Lewis acidity, which may enhance Cas12a activity by stabilizing the transition state geometry.^[^ ^28^, ^29^ ^]^

### 2.4 Detection Performance of Cas12a@hMNS Nanoprobe In Vitro

**Figure 3:**
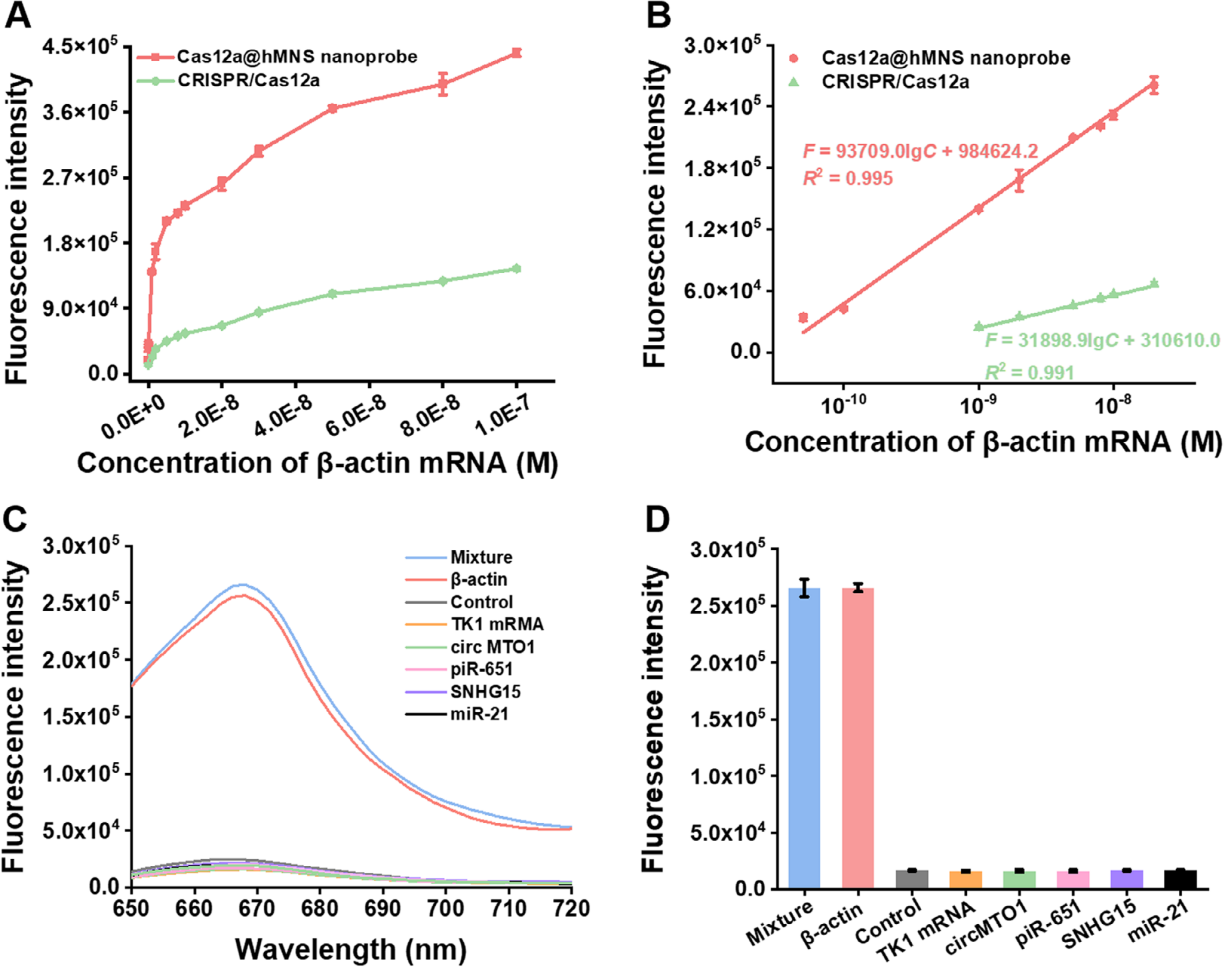
A) Fluorescence intensities of Cas12a@hMNS nanoprobe (red curve) and free CRISPR/Cas12a system (green curve) in response to different concentrations of β‐actin mRNA. B) Linear correlation of the fluorescence intensity produced by the Cas12a@hMNS nanoprobe (red curve) and free CRISPR/Cas12a system (green curve) with the logarithm of β‐actin mRNA concentration. C) Fluorescence emission spectrum in response to the mixture of β‐actin mRNA + all interreference RNAs, 10 nM β‐actin mRNA, 10 nM TK1 mRNA, 10 nM circMTO1, 10 nM piR‐651, 10 nM SNHG15, 10 nM miR‐21, and the control group, respectively. D) Measurement of fluorescence intensity induced by the mixture of β‐actin mRNA + all interfering RNAs, 10 nM β‐actin mRNA, 10 nM TK1 mRNA, 10 nM circMTO1, 10 nM piR‐651, 10 nM SNHG15, 10 nM miR‐21, and the control group, respectively. Data are presented as mean ± SD (*n* = 3).

Under the optimized conditions (Figures S4–S9, Supporting Information), we examined the sensitivity of this Cas12a@hMNS nanoprobe. The Cy5 fluorescence intensity (*F*) enhances gradually with the increasing β‐actin mRNA concentration (*C*) (Figure 3A, red curve; Figure S10, Supporting Information), and it is linearly dependent on the logarithmic value of β‐actin mRNA concentration from 5 × 10^−11^ to 2 × 10^−8^ M (Figure 3B, red curve). The regression equation is *F* = 984 624.2 + 93 709.0 lg *C* (*R* ^2^ = 0.995), with an LOD of 63.6 pM. To highlight the contribution of Mn^2+^ generated by GSH‐mediated degradation of hMNS, we investigated the sensitivity of free CRISPR/Cas12a system for comparison. The Cy5 fluorescence intensity (*F*) enhances slowly with the increasing β‐actin mRNA concentration (C) (Figure 3A, green curve; Figure S11, Supporting Information), and it is linearly correlated with the logarithmic concentration of β‐actin mRNA in the dynamic range of 1 × 10^−9^ — 2 × 10^−8 ^M (Figure 3B, green curve). The regression equation is *F* = 310 610.0 + 31 898.9 lg *C* (*R* ^2^ = 0.991), and the limit of detection (LOD) is measured to be 748 pM. Notably, the dynamic range of the Cas12a@hMNS nanoprobe is 10‐fold larger than that of the free CRISPR/Cas12a system, and the detection limit of the Cas12a@hMNS nanoprobe (63.6 pM) is 11.8‐fold lower than that of the free CRISPR/Cas12a system (748 pM). The improved sensitivity of the Cas12a@hMNS nanoprobe may be attributable to its high signal gain and high signal‐to‐noise ratio.

The selectivity of this Cas12a@hMNS nanoprobe toward β‐actin mRNA is assessed by using TK1 mRNA, circMTO1, piRNA‐651, lncRNA SNHG15, and miRNA‐21 as the interfering RNAs. As depicted in Figure 3C,D, only target β‐actin mRNA can induce a high Cy5 fluorescence response, which can be well distinguished from those induced by interfering RNAs (i.e., TK1 mRNA, circMTO1, piRNA‐651, lncRNA SNHG15, and miRNA‐21) and the control group without any RNAs, suggesting that only β‐actin mRNA can activate Cas12a/crRNA to initiate the *trans*‐cleavage of reporters. Moreover, identical Cy5 fluorescence responses are generated from β‐actin mRNA and the mixture of β‐actin mRNA + all interfering RNAs, indicating the good selectivity of this nanoprobe toward β‐actin mRNA.

**Figure 4:**
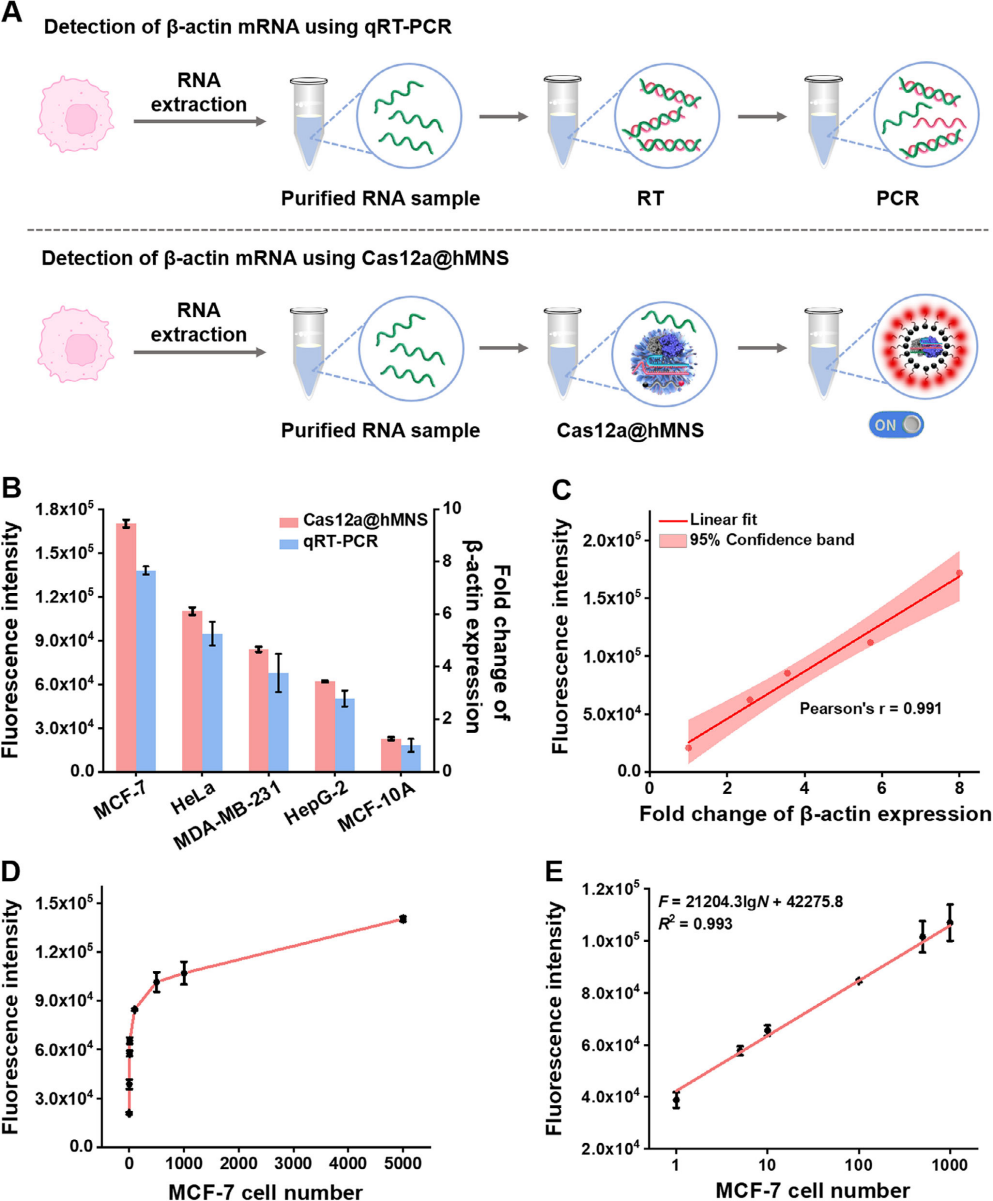
A) Schematic diagram of endogenous mRNA analysis by the Cas12a@hMNS nanoprobe and qRT‐PCR. B) Fluorescence intensities and fold changes in response to MCF‐7, HeLa, MDA‐MB‐231, HepG‐2, and MCF‐10A cells. C) Correlation analysis between the Cas12a@hMNS nanoprobe and qRT‐PCR for the measurement of β‐actin mRNA expression in various cells. D) Variance of fluorescence intensity induced by varying numbers of MCF‐7 cells. E) Fitting curve of fluorescence intensity against MCF‐7 cell numbers. Data are presented as mean ± SD (*n* = 3).

To investigate the performance of this Cas12a@hMNS nanoprobe for endogenous β‐actin mRNA analysis, we measured β‐actin mRNA expressions in MCF‐7, HeLa, MDA‐MB‐231, HepG‐2, and MCF‐10A cells (**Figure** **4**A). The fluorescence responses generated by MCF‐7, HeLa, MDA‐MB‐231, and HepG‐2 cells are greatly higher than that generated by MCF‐10A cells (Figure 4B, red columns), demonstrating that β‐actin mRNA is upregulated in tumor cells.^[^ ^47^, ^48^, ^49^ ^]^ These results are supported by quantitative reverse transcription polymerase chain reaction (qRT‐PCR) (Figure 4B, blue columns), and the expression of β‐actin mRNA in different cells measured by the Cas12a@hMNS nanoprobe reveals a strong correlation with those measured by qRT‐PCR (Pearson′s r = 0.991, Figure 4C). In addition, we monitored the fluorescence response induced by varying numbers of MCF‐7 cells. Figure 4D shows the variance of fluorescence response with the MCF‐7 cell numbers. The fluorescence intensity (*F*) increases linearly with the logarithm of MCF‐7 cell numbers (*N*) over the range of 1–1000 (Figure 4E), with a regression equation of *F* = 42 275.8 + 21 204.3 lg *N* (*R* ^2^ = 0.993). The calculated LOD is 1 cell. These results clearly demonstrate that this Cas12a@hMNS nanoprobe enables accurate measurement of endogenous β‐actin mRNA with single‐cell sensitivity.

**Figure 5:**
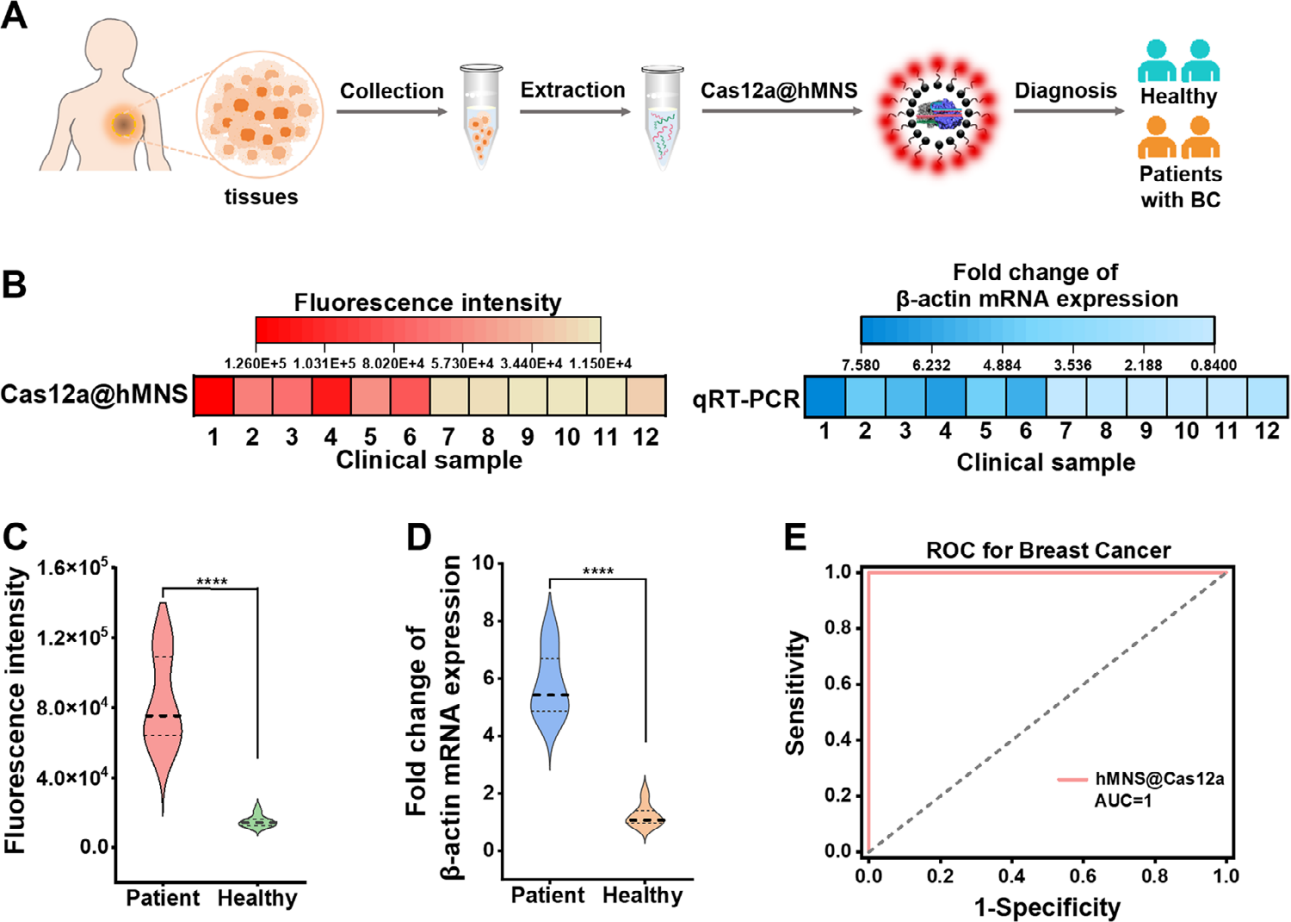
A) Schematic diagram for β‐actin mRNA profiling in breast cancer tissues from their healthy counterparts. B) Profiling of β‐actin mRNA in breast cancer tissues (1–6) and their healthy counterparts (7– 12) by the Cas12a@hMNS nanoprobe and qRT‐PCR, respectively. C) Violin plot of fluorescence intensities resulting from breast cancer tissues and their healthy counterparts determined by the Cas12a@hMNS nanoprobe. D) Violin plot of fold change of β‐actin mRNA expression resulting from breast cancer tissues and their healthy counterparts determined by qRT‐PCR. ^****^ *p* < 0.0001, calculated by unpaired, two‐tailed Student's *t*‐test. E) ROC analysis of the Cas12a@hMNS nanoprobe detection accuracy in clinical applications.

The practicality of this Cas12a@hMNS nanoprobe in clinical diagnosis is evaluated by profiling the β‐actin mRNA levels in breast cancer tissues (1–6) and their healthy counterparts (7–12) (**Figure** **5**A). The fluorescence intensities generated by breast cancer tissues are much higher than those generated by their healthy counterparts (Figure 5B), and all breast cancer tissues exhibit higher levels of β‐actin mRNA than their healthy counterparts (*p* < 0.0001) (Figure 5C), implying that the expression of β‐actin mRNA is upregulated in breast cancer.^[^ ^49^ ^]^ The qRT‐PCR also confirms a significant difference in β‐actin mRNA level between breast cancer tissues and their healthy counterparts (*p* < 0.0001) (Figure 5B,D). Moreover, we introduced receiver operating characteristic (ROC) curve analysis to verify the discrimination accuracy of β‐actin mRNA level between breast cancer patients and healthy counterparts. The ROC curve of the Cas12a@hMNS nanoprobe exhibits an area under the curve (AUC) of 1.0 for breast cancer tissues compared with their healthy counterparts, with a sensitivity and specificity of 100% (Figure 5E). The above results prove that this Cas12a@hMNS nanoprobe can accurately discriminate breast cancer patients from healthy counterparts, holding great promise in cancer diagnosis.

### 2.5 In Vivo Imaging Performance of Cas12a@hMNS Nanoprobe

Toxicity is a critical consideration for live‐cell imaging of the Cas12a@hMNS nanoprobe. We evaluated the potential cytotoxicity of this Cas12a@hMNS nanoprobe toward MCF‐7 cells using cell viability analysis. After incubation with different concentrations of Cas12a@hMNS nanoprobe (up to 60 µg mL^−1^ hMNS) for 24 h, the viability of MCF‐7 cells maintains more than 80% (Figure S12, Supporting Information), indicating that this Cas12a@hMNS nanoprobe exhibits low toxicity at working concentrations. Moreover, excess intracellular Mn^2+^ can be efficiently effluxed by transport membrane proteins, preventing accumulation beyond physiological levels.^[^ ^50^, ^51^ ^]^ Subsequently, the lysosomal escape of Cas12a@hMNS is confirmed through colocalization assay, and commercial Lipofectamine 3000 is employed as a reference carrier for comparison (Figure S13, Supporting Information). Lysosomes are stained with LysoTracker Green (green), and nuclei are stained with Hoechst 33 342 (blue). Experimental evidence demonstrates a notable lysosomal escape efficiency of Cas12a@hMNS with a lower Pearson's correlation coefficient of 0.27. Interestingly, the Lipofectamine complexes exhibit much lower lysosomal escape efficiency with a Pearson's correlation coefficient of 0.48, highlighting the essential role of hMNS in facilitating the endosomal escape process.

**Figure 6:**
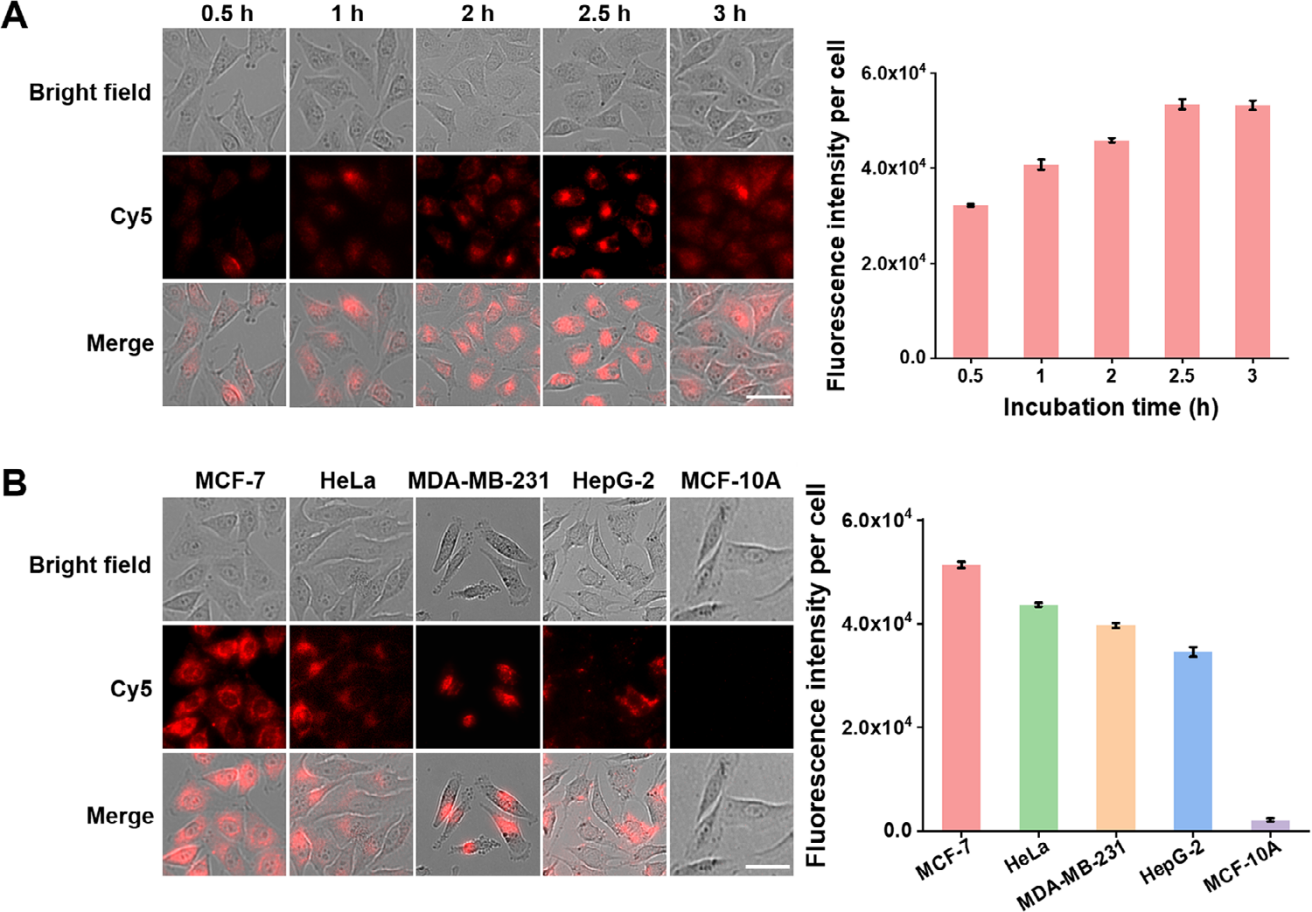
A) Fluorescence imaging of MCF‐7 cells treated with the Cas12a@hMNS nanoprobe for different time intervals and the corresponding statistics of mean fluorescence intensity. B) Fluorescence imaging of MCF‐7, HeLa, MD‐MBA‐231, HepG‐2, and MCF‐10A cells after treatment with the Cas12a@hMNS nanoprobe and the corresponding statistics of mean fluorescence intensity. Scale bar: 25 µm. Data are presented as mean ± SD (*n* = 3).

The hMNS nanocarrier is able to efficiently deliver the CRISPR/Cas12a‐based sensing system into MCF‐7 cells. After incubation with the Cas12a@hMNSnanoprobe, a time‐dependent enhancement in Cy5 fluorescence is detected in MCF‐7 cells, and the fluorescence signal reaches the maximum value at 2.5 h (**Figure** **6**A). Thus, the incubation time of 2.5 h is used in the subsequent experiments. To prove the capability of the Cas12a@hMNS nanoprobe for distinguishing different cell lines, we selected four tumor cells (i.e., MCF‐7, HeLa, MD‐MBA‐231, and HepG‐2 cells) and one normal cell (i.e., MCF‐10A cells) as the models. As depicted in Figure 6B, no detectable fluorescence signal is observed in normal MCF‐10A cells. In contrast, strong fluorescence signals are obtained in cancer cell lines, including MCF‐7, HeLa, MD‐MBA‐231, and HepG‐2 cells, suggesting that the fluorescence signal results from β‐actin mRNA‐induced activation of the CRISPR/Cas12a‐based sensing system. These results demonstrate that this Cas12a@hMNS nanoprobe enables precise imaging of β‐actin mRNA with tumor‐cell specificity.

**Figure 7:**
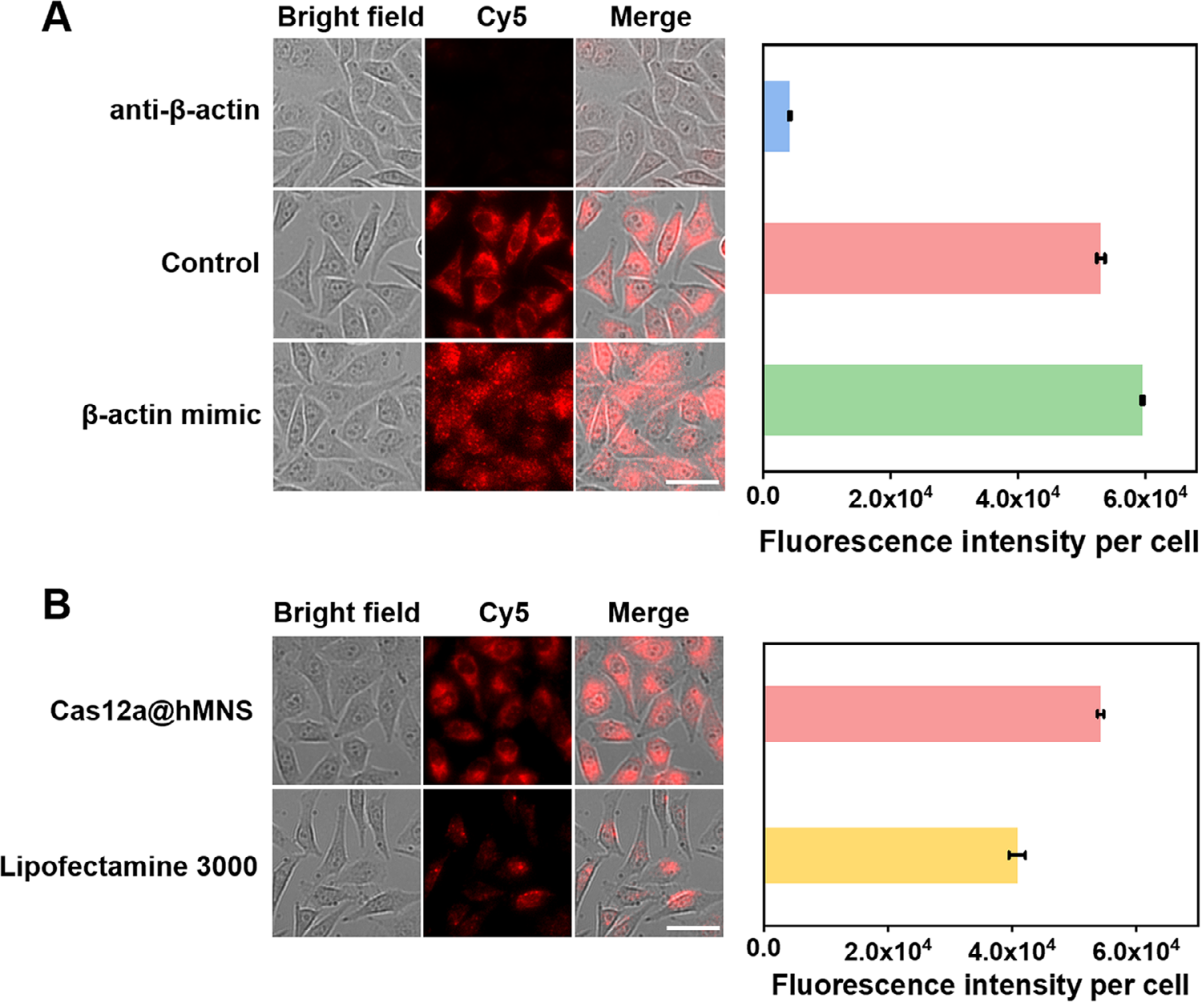
A) Fluorescence imaging of MCF‐7 cells after treatment with β‐actin mRNA mimic (up‐regulation) and anti‐β‐actin mRNA (down‐regulation), and the corresponding statistics of mean fluorescence intensity. B) Intracellular β‐actin mRNA analysis using hMNS and commercial liposome for the delivery of CRISPR/Cas12a‐based sensing system. Scale bar: 25 µm. Data are presented as mean ± SD (*n* = 3).

Due to the different abundance of β‐actin mRNA in cells at different stages, the capability of the Cas12a@hMNS nanoprobe for discriminating the varied expression of β‐actin mRNA in living cells is valuable for studying the physiological processes and pathological mechanisms of cancer. β‐actin mRNA mimic and anti‐β‐actin mRNA oligonucleotides complementary to target mRNA are introduced into MCF‐7 cells to upregulate and downregulate the expression level of endogenous β‐actin mRNA, respectively. As depicted in **Figure** **7**A, the fluorescence intensity of MCF‐7 cells treated with β‐actin mRNA mimic is stronger than that of the untreated MCF‐7 cells (control), indicating target‐dependent activation of the Cas12a@hMNS nanoprobe. The treatment of MCF‐7 cells with anti‐β‐actin mRNA oligonucleotide induces a significant decrease in fluorescence intensity compared with the untreated MCF‐7 cells due to efficient knockdown of target β‐actin mRNA. These results suggest that the Cas12a@hMNS nanoprobe facilitates the specific monitoring of the dynamic change of β‐actin mRNA in living cells.

We compared the efficacies of hMNS (Scheme 1B) and Lipofectamine 3000 (Scheme 1A) as the carriers of CRISPR/Cas12a system for live‐cell imaging. As depicted in Figure 7B, different from Lipofectamine 3000 that induces a lower fluorescence intensity in MCF‐7 cells, Cas12a@hMNS nanoprobe generates a 1.33‐fold higher fluorescence intensity in MCF‐7 cells, suggesting that hMNS is superior to commercial liposome carriers. Notably, hMNS exhibits three key advantages: 1) efficient intracellular delivery of the CRISPR/Cas12a system, 2) fast payload release, and 3) Mn^2^⁺‐enhanced signal amplification. The Cas12a@hMNS nanoprobe remains stable in the extracellular environment with low GSH concentration (<20 µm) but rapidly internalizes into cells due to the 100‐fold higher intracellular GSH concentration (≈2 mM)^[^ ^39^ ^]^). Moreover, high concentrations of endogenous GSH can induce the complete degradation of hMNS within a short period of time to release the CRISPR/Cas12a system for immediate intracellular mRNA imaging. In addition, GSH‐catalyzed reduction of hMNS can produces abundant Mn^2+^ as an accelerator to achieve self‐sufficiency of CRISPR/Cas12a‐mediated *trans*‐cleavage of reporters, inducing a significantly amplified fluorescence signal.

**Figure 8:**
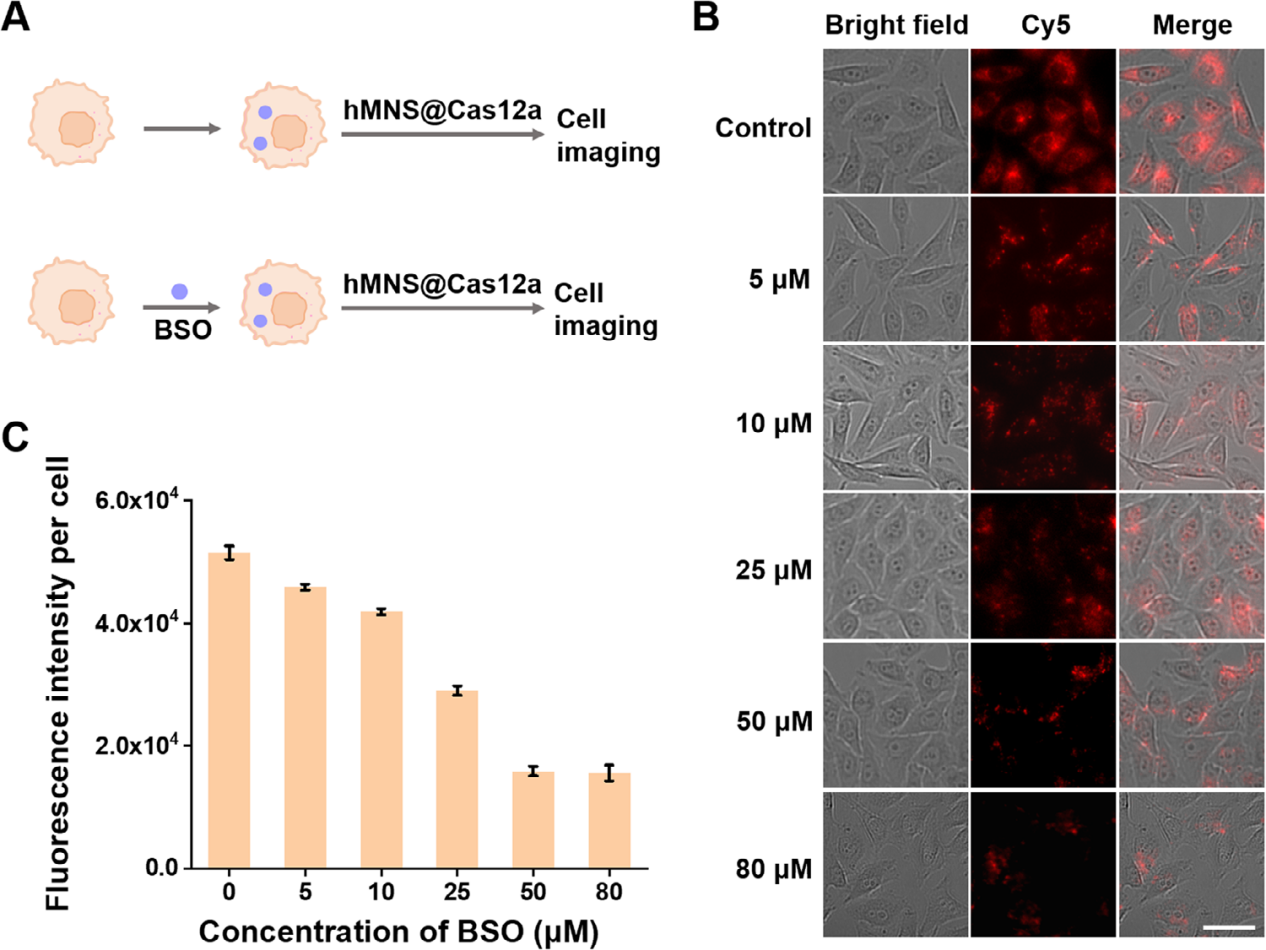
A) Schematic of cellular inhibitor treatment. B) Fluorescence imaging of MCF‐7 cells treated with different‐concentration BSO. C) Statistics of mean fluorescence intensity in (B). Scale bar: 25 µm. Data are presented as mean ± SD (*n* = 3).

GSH‐induced degradation of hMNS in living cells can rapidly release the CRISPR/Cas12a‐based sensing system and simultaneously provide adequate Mn^2+^ to improve the *trans*‐cleavage activity of Cas12a. In order to validate the essential role of endogenous GSH for Cas12a@hMNS nanoprobe, we pretreated MCF‐7 cells with buthionine sulfoximine (BSO, a potent GSH synthase inhibitor) to deplete GSH.^[^ ^52^ ^]^ As depicted in **Figure** **8**, compared with the control cells without BSO treatment, MCF‐7 cells treated with BSO show a dose‐dependent decrease in fluorescence signal, followed by leveling off at the BSO concentration of 50 µm, suggesting the critical role of GSH in the activation of the Cas12a@hMNS nanoprobes.

## 3 Conclusion

In summary, we have engineered a Cas12a@MnO_2_ nanosponge (hMNS) nanoprobe that synergistically integrates nanocarrier delivery with kinetic enhancement for efficient imaging of mRNA in living cells. In this assay, the multi‐functional hMNS not only serves as a biodegradable carrier for delivering CRISPR/Cas12a sensing components into living cells, but also as a Mn^2+^ donor for supplying adequate Mn^2+^ in the GSH‐rich tumor microenvironment (TME) to overcome the kinetic barrier of conventional CRISPR/Cas12a‐based in vivo RNA sensing system. Notably, hMNS offers two key advantages over the commercial CRISPR/Cas12a carriers: 1) facile CRISPR/Cas12a loading and 2) superior imaging efficiency in living cells. All components of the CRISPR/Cas12a‐based sensing system can be directly adsorbed onto the hMNS surface through a simple one‐step co‐assembly strategy, enabling synchronous internalization of these components into living cells. In comparison with commercial liposome carriers, hMNS can induce much higher fluorescence intensity due to its distinct advantages of efficient cellular uptake, rapid payload release, and accelerated signal amplification kinetics. The constructed Cas12a@hMNS nanoprobe enables preamplification‐free detection of β‐actin mRNA with single‐cell sensitivity, and differentiation of β‐actin mRNA expression between breast cancer tissues and their healthy counterparts. Furthermore, it can be applied for real‐time tracking of β‐actin mRNA dynamics in living cells and visual discrimination of tumor cells from normal cells. Importantly, this Cas12a@hMNS nanoprobe can be modularly adaptable for imaging a variety of intracellular biomolecules (e.g., ncRNAs, enzymes, and small molecules), opening a promising avenue for clinical diagnostics and precision medicine.

## 4 Experimental Section

### Construction of the Cas12a@hMNS Nanoprobe

The Cas12a@hMNS nanoprobe was prepared by mixing 2.5 µg mL^−1^ hMNS with 10 nM crRNA/EB‐activator and 200 nM reporter, and incubation at room temperature for 20 min, followed by adding 10 µL of Tris‐HAc buffer (100 mM, pH 7.4). Then, 50 nM Cas12a was added to the above mixture and incubated for 10 min at room temperature to obtain the Cas12a@hMNS nanoprobe.

### In Vitro Detection of mRNA

The obtained Cas12a@hMNS nanoprobe was incubated with 0.5 mM GSH for 2 min at room temperature. Then, different concentrations of β‐actin mRNA and 1 × NEBuffer 2.1 (50 mM NaCl, 10 mM Tris‐HCl, 10 mM MgCl_2_, 10 µg mL^−1^ BSA, pH 7.9) were added into the solution with a total volume of 20 µL, and then incubated at 37 °C for 60 min.

### Fluorescence Measurements

The fluorescence intensity of the reaction products was measured by using a FLS‐1000 fluorescence spectrometer (Edinburgh Instruments, Livingston, U.K.) with an excitation wavelength of 635 nm. The emission spectra were recorded over the wavelength range of 650–750 nm with a slit width of 6 nm for both excitation and emission. The fluorescence intensity at 668 nm was used for data analysis.

### The qRT‐PCR Assay

Total RNA obtained from different cell lines, breast cancer tissues, and their healthy counterparts was reverse transcribed to cDNA using the Evo M‐MLV RT mix kit with gDNA Clean for qPCR (Accurate Biotechnology, Hunan, China) according to the manufacturer's instructions. Then, the β‐actin mRNA levels were quantified by RT‐PCR using SYBR Green Premix Pro Taq HS qPCR kit (Accurate Biotechnology, Hunan, China) in a BIO‐RAD CFX connect real‐time system. The results were analyzed by using the 2^−∆∆Ct^ method with MCF‐10A cells and healthy counterparts as the control.

### Imaging of mRNA in Living Cells

All cell lines were seeded in 35‐mm^2^ confocal dishes and cultured for 12 h until the cell density reached 80–85%. After the dish was washed three times with PBS, 1 mL of Opti‐MEM containing Cas12a@hMNS nanoprobe was added to the dish and incubated at 37 °C for 2.5 h. The final amount of each component was 50 µL of hMNS (100 µg mL^−1^), 0.6 µL of crRNA/EB‐activator (5 µm), 0.6 µL of reporter (100 µm), and 1.5 µL of cas12a (10 µm). Then, the cells were washed thoroughly with PBS and replenished with fresh Opti‐MEM medium. An Olympus IX71 microscope with a 10 × objective was used for intracellular fluorescence imaging.

### Statistical Analysis

Statistical analysis of the experimental data was conducted via OriginPro 2021. All experiments were performed in at least three independent replicates, and data are presented as mean ± standard deviation (Mean ± SD). An unpaired, two‐tailed Student's *t*‐test was applied for comparisons between two groups. A *p*‐value of less than 0.05 was considered statistically significant.

### Ethical Approval Statement

Ethical approval for the clinical sample analysis experiments with human participants was reviewed and approved by lEC for Clinical Research of Zhongda Hospital, Affiliated to Southeast University (2025ZDKYSB346). All participants or their legally authorized representatives provided written informed consent prior to participation. The consent forms explicitly permitted the use of the resected tissue for future research purposes.

## Conflict of Interest

The authors declare no conflict of interest.

## Author Contributions

W.L. and L.W. contributed equally to this work. W.L. designed the experiments and wrote the initial manuscript. L.W. performed the majority of the experiments and analyzed the data. F.M. and C.Z. supervised the project and revised the manuscript.

## Supporting information



Supporting Information

## Data Availability

The data that support the findings of this study are available from the corresponding author upon reasonable request.
