# Supplementary material for: MnO2 Nanosponge‐Accelerated Cas12a Trans‐Cleavage: Breaking the Kinetic Barrier for In Vivo RNA Imaging
Source: Adv Sci (Weinh). 2025 Oct 14;12(48):e11942. doi: 10.1002/advs.202511942 (PMC12752570; doi:10.1002/advs.202511942)
Supplement: Supplementary file 1 — Supporting Information [file ADVS-12-e11942-s001.docx]

Supporting Information

**MnO_2_ Nanosponge-Accelerated Cas12a *Trans*-Cleavage: Breaking the Kinetic Barrier for In Vivo RNA Imaging**

Wen-jing Liu,^[a]^ Lu-yao Wang,^[a]^ Fei Ma,*^[a]^ and Chun-yang Zhang*^[a]^

[a] School of Chemistry and Chemical Engineering, State Key Laboratory of Digital Medical Engineering, Southeast University, Nanjing 211189, China.

* Corresponding authors: E-mail: [fei@seu.edu.cn](mailto:fei@seu.edu.cn), [zhangcy@seu.edu.cn](mailto:zhangcy@seu.edu.cn).

**Table of Contents**

[Materials and chemicals S3](#_Toc208495274)

[Preparation of honeycomb MnO_2_ nanosponge (hMNS) S6](#_Toc208495275)

[Gel electrophoresis analysis S6](#_Toc208495276)

[Cell culture and total RNA extraction from cells and tissues S7](#_Toc208495277)

[Evaluation of cellular toxicity of Cas12a@hMNS S7](#_Toc208495278)

[Colocalization analysis S8](#_Toc208495280)

[Working mechanism and detailed sequences of CRISPR/Cas12a-based sensing system S9](#_Toc208495281)

[Color change of solution before and after the decomposition of hMNS by GSH S10](#_Toc208495282)

[Real-time fluorescence monitoring S11](#_Toc208495283)

[Optimization of the concentration of hMNS S12](#_Toc208495284)

[Optimization of the concentration of GSH S13](#_Toc208495285)

[Optimization of the concentration of crRNA/EB-activator S14](#_Toc208495286)

[Optimization of the concentration of Cas12a S15](#_Toc208495287)

[Optimization of the concentration of reporter S16](#_Toc208495288)

[Optimization of the reaction time S17](#_Toc208495289)

[Fluorescence emission spectrum of Cas12a@hMNS nanoprobe S18](#_Toc208495290)

[Fluorescence emission spectrum of free CRISPR/Cas12a system S19](#_Toc208495291)

[Cytotoxicity assay S20](#_Toc208495292)

[Colocalization assay S21](#_Toc208495293)

[References S22](#_Toc208495294)

**Materials and Methods**

**Materials and chemicals**

All oligonucleotides (see Table S1) were synthesized and HPLC purified by Accurate Biotechnology (Changsha, China). Lba Cas12a (cpf1) was obtained from Huicheng Biotechnology Co. Ltd. (Shanghai, China). 10 × NEBuffer 2.1 (500 mM NaCl, 100 mM Tris-HCl, 100 mM MgCl_2_, 100 μg/ml BSA, pH 7.9) was obtained from New England Biolabs (Ipswich, MA, USA). Potassium permanganate (KMnO_4_), oleic acid (OA) and manganese (II) chloride were bought from Aladdin Biochemical Technology Co., Ltd. (Shanghai, China). Glutathione (GSH) and diethylpyrocarbonate (DEPC)-treated water (RNase free) were purchased from Sangon Biotech Co. Ltd. (Shanghai, China). L-Buthionine-sulfoximine (BSO) was obtained from Mackin Biochemical Technology Co., Ltd. (Shanghai, China). SYBR Gold was bought from Life Technologies (Carlsbad, CA, USA). Lipofectamine™ 3000 transfection reagent was bought from Thermo Fisher Scientific (Massachusetts, USA). Human breast cancer cell lines (MCF-7 cells and MDA-MB-231 cells), human breast normal cell lines (MCF-10A cells), human cervical cancer cell lines (HeLa cells) and human hepatocellular carcinoma cell lines (HepG2) were bought from Cell Bank of Chinese Academy of Sciences (Shanghai, China). The formalin-fixed paraffin-embedded (FFPE) breast tissue sections were obtained from Zhongda Hospital, Affiliated to Southeast University (Nanjing, China), and the research was approved by lEC for Clinical Research of Zhongda Hospital, Affiliated to Southeast University (2025ZDKYSB346).

**Table S1.** Sequences of the oligonucleotides

| **Oligonucleotides** | **Sequences (5′-3′)** |
| --- | --- |
| crRNA | UAA UUU CUA CUA AGU GUA GAU GCA UCU CUA CAU ACU CAG AC |
| β-actin mRNA | CCC AGC CAU GUA CGU UGC UA |
| Reporter | Cy5-TTT TTT-BHQ2 |
| Activator | GTC TGA GTA TGT AGA GAT GC |
| EB-3 | GTC TGA GTA TGT AGA GAT GCA TC |
| EB-6 | GTC TGA GTA TGT AGA GAT GCA TCT AC |
| EB-7 | GTC TGA GTA TGT AGA GAT GCA TCT ACA |
| EB-8 | GTC TGA GTA TGT AGA GAT GCA TCT ACA C |
| EB-9 | GTC TGA GTA TGT AGA GAT GCA TCT ACA CT |
| EB-11 | GTC TGA GTA TGT AGA GAT GCA TCT ACA CTT A |
| EB-13 | GTC TGA GTA TGT AGA GAT GCA TCT ACA CTT AGT |
| EB-15 | GTC TGA GTA TGT AGA GAT GCA TCT ACA CTT AGT AG |
| EB-17 | GTC TGA GTA TGT AGA GAT GCA TCT ACA CTT AGT AGA A |
| EB-19 | GTC TGA GTA TGT AGA GAT GCA TCT ACA CTT AGT AGA AAT |
| EB-21 | GTC TGA GTA TGT AGA GAT GCA TCT ACA CTT AGT AGA AAT TA |
| EB-mRNA | GTC TGA GTA TGT AGA GAT GCA TCT ACA CGT ACA TGG CTG GG |
| TK1 mRNA | CUG GUG AUC AAG UAU GCC AAA GAC ACU CGC UAC AGC AGC A |
| LncRNA SNHG15 | UGC CUG CCA UCC GUC AGU GUU UC |
| circMTO1 | GUG GGG UUG UUU UGG GUC AGA UGU CAU GUA |
| miRNA-21 | UAG CUU AUC AGA CUG AUG UUG A |
| piRNA-651 | AGA GAG GGG CCC GUG CCU UGG AAA GCG UC |
| Anti-β-actin | TAG CAA CGT ACA TGG CTG GG |
| β-actin-forward primer | ATG GAT GAC GAT ATC GCT GC |
| β-actin-reverse primer | CTT CTG ACC CAT ACC CAC CA |
| GAPDH-forward primer | ATC TCT GCC CCC TCT GCT GA |
| GAPDH- reverse primer | GAT GAC CTT GCC CAC AGC CT |

**P****reparation of honeycomb MnO_2_ nanosponge (hMNS)**

The hMNS was synthesized according to previously reported methods. ^[1, 2]^ First, 0.1 g of KMnO_4_ was dissolved in 50 mL of deionized water and vigorously stirred for 30 min. Second, 1 mL of oleic acid (OA) was added to the above solution and vigorously stirred for 5 h. Third, the product was centrifuged at 10,000 rpm for 30 min, and then washed with water and alcohol for three times, respectively. Subsequently, the bulk manganese dioxide was dried in a drying oven at 60 ℃ and stored in a centrifuge tube. To obtain the MnO_2_ nanosponge, 10 mg of bulk manganese dioxide was dispersed in 20 mL of deionized water and ultrasonicated for at least 10 h, followed by centrifugation at 2000 rpm for 30 min. Finally, the resultant supernatant was stored at 4 ℃ for subsequent experiments.

**Gel electrophoresis analysis**

The β-actin mRNA-induced activation of crRNA was characterized by 14% nondenaturing PAGE in 1× TBE buffer (9 mM Tris-HCl, 9 mM boric acid, 0.2 mM EDTA, pH 7.9) at a constant voltage of 110 V for 55 min with 1 × SYBR Gold as the fluorescent indicator. The CRISPR/Cas12a-mediated trans-cleavage products were analyzed by using 14% nondenaturing PAGE in 1 × TBE buffer (9 mM Tris-HCl, 9 mM boric acid, 0.2 mM EDTA, pH 7.9) at a constant voltage of 110 V for 50 min with 1 × SYBR Gold as the fluorescent indicator. Subsequently, the gels were imaged by using a Bio-Rad ChemiDoc MP Imaging System (Hercules, California, USA).

Cell culture and total RNA extraction from cells and tissues

MCF-7, MDA-MB-231, HeLa and HepG-2 cells were cultured in Dulbecco’s modified Eagle’s medium (DMEM, Invitrogen, USA) containing 10% FBS and 1% PS. MCF-10A cells were grown in complete S-7 growth medium (Procell Life Science & Technology Co., Ltd.). All the cells were incubated in a 100% humidified chamber containing 5% CO_2_ at 37 °C. Prior to the extraction, the numbers of cells were counted by a Countstar automated cell counter (IC 1000, Inno-Alliance Biotech Inc., Wilmington, DE, USA). The SanPrep column microRNA extraction kit (Sangon Biotech, Shanghai) was employed to obtain the cellular total RNA extracts. The miRNeasy FFPE kit was used to obtain the total RNA extracts from the breast cancer tissue samples and their healthy counterparts. The concentration of total RNA was determined by a NanoDrop 2000c spectrophotometer (Thermo Scientific, Wilmington, Delaware, USA).

**Evaluation of cellular toxicity of Cas12a@hMNS**

The potential cytotoxicity of Cas12a@hMNS in MCF-7 cells were evaluated by MTT assays. MCF-7 cells were seeded in 96-well plates for 12 h and then treated with different concentrations of Cas12a@hMNS (1 – 60 μg/mL) in Opti-MEM at 37 °C for 24 h. After washing with PBS, the cell growth medium was replaced by fresh medium containing 10% CCK-8 and incubated at 37 °C for another 1 h. Finally, the cell viability was determined by measuring the optical density (OD) at 490 nm using a microplate reader.

**Colocalization analysis**

MCF-7 cells were seeded into confocal dishes for 12 h and discard the medium. 1 mL of fresh medium and Cas12a@hMNS (assembled by Cas12a, Cy5-labeled crRNA, and hMNS) or Lipofectamine complexes (assembled by Lipofectamine 3000, Cas12a, and Cy5-labeled crRNA) were added and incubated at 37 °C for 2.5 h. Subsequently, all groups were incubated with a fresh medium containing 50 nM LysoTracker green for 30 min. Finally, the cells were stained by Hoechst 33342 (5 μg/mL) for another 15 min using a standard procedure, and imaged by using an Olympus IX71 microscope with a 40 × objective.

**Supplementary results**

**Working mechanism and detailed sequences of CRISPR/Cas12a-based sensing system**


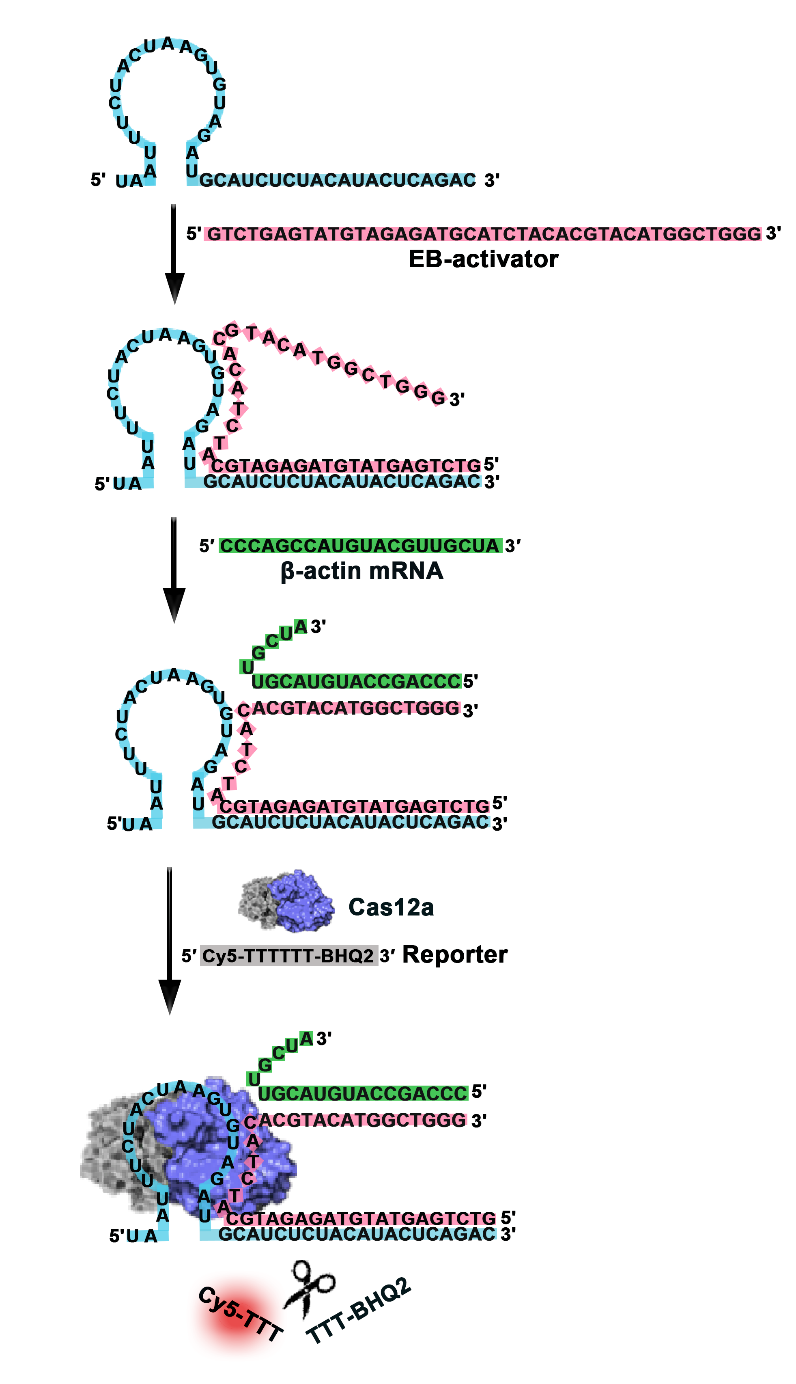


**Figure S1.** Working mechanism and detailed sequences of CRISPR/Cas12a-based sensing system for mRNA detection.

**Color change of solution before and after the decomposition of hMNS by GSH**


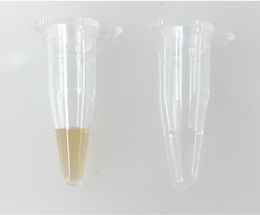


**Figure S2.** The images of the Cas12a@hMNS nanoprobe and Cas12a@hMNS nanoprobe + GSH. After the decomposition of hMNS, the solution color was changed from brown (left) to clear (right).

**Real-time fluorescence monitoring**


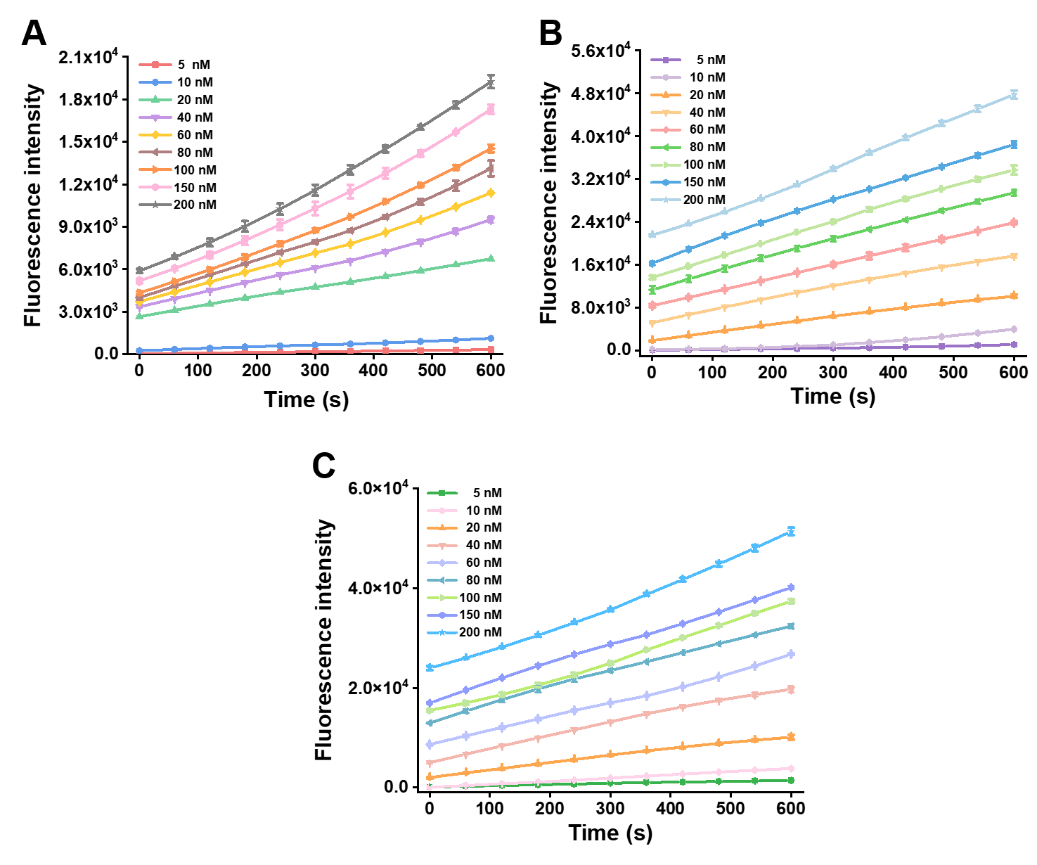


**Figure S3.** (A) Real-time monitoring of fluorescence intensity in response to different-concentration reporter from 5 to 200 nM in the absence of Mn^2+^. (B) Real-time monitoring of fluorescence intensity in response to different-concentration reporter from 5 to 200 nM in the presence of Mn^2+^. (C) Real-time monitoring of fluorescence intensity in response to different-concentration reporter from 5 to 200 nM in the presence of hMNS. Data are presented as mean ± SD (*n* = 3).

**Optimization of the concentration of hMNS**

In order to achieve the best assay performance, we optimized a series of experimental parameters including the concentration of hMNS, the concentration of GSH, the concentration of crRNA/EB-activator, the concentration of Cas12a, the concentration of reporter, and the reaction time. We used the *F*/*F*_0_ value to optimize the experimental parameters, where *F* and *F*_0_ are the fluorescence intensity in the presence and absence of β-actin mRNA, respectively.

As the nanocarrier and donor of Mn^2+^, hMNS is a crucial component in the Cas12a@hMNS nanoprobe, and it has a significant effect on the operational efficiency of Cas12a@hMNS nanoprobe. We optimized the concentration of hMNS (Figure S4). The *F*/*F*_0_ value enhances with the increasing concentration from 1 to 2.5 ng/µL, followed by the decrease beyond the concentration of 2.5 ng/µL. Thus, 2.5 ng/µL hMNS is used in the subsequent researches.


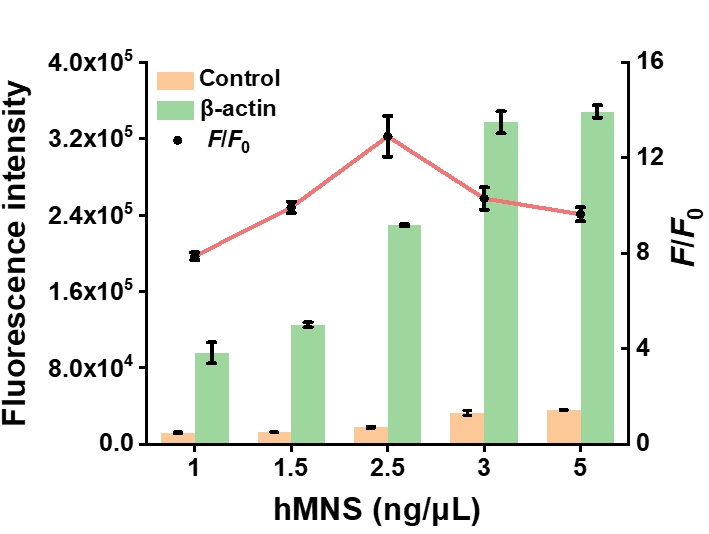


**Figure S4.** Variance of the *F*/*F*_0_ value with different concentrations of hMNS. Data are presented as mean ± SD (*n* = 3).

**Optimization of the concentration of GSH**

In this assay, the concentration of GSH may directly influence the yield of Mn^2+^ and affect the *trans* cleavage efficiency of Cas12a. As shown in Figure S5, when the GSH concentration increases from 0.05 to 0.5 mM, the *F*/*F*_0_ value enhances correspondingly, followed by the decrease beyond the concentration of 0.5 mM. Thus, 0.5 mM GSH is used in the subsequent researches.


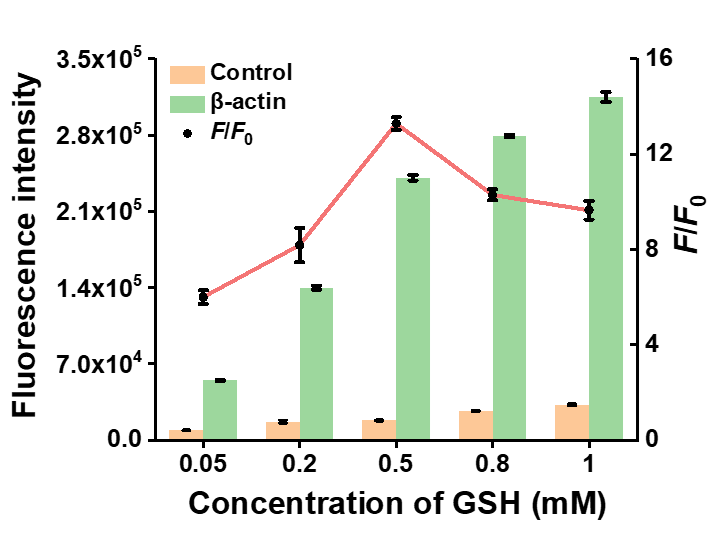


**Figure S5.** Variance of the *F*/*F*_0_ value with different concentrations of GSH. Data are presented as mean ± SD (*n* = 3).

**Optimization of the concentration of crRNA/EB-activator**

We optimized the concentration of crRNA/EB-activator. As shown in Figure S6, when the crRNA/EB-activator concentration increases from 5 to 10 nM, the *F*/*F*_0_ value improves correspondingly, followed by the decrease beyond the concentration of 10 nM. Thus, 10 nM crRNA/EB-activator is used in the subsequent researches.


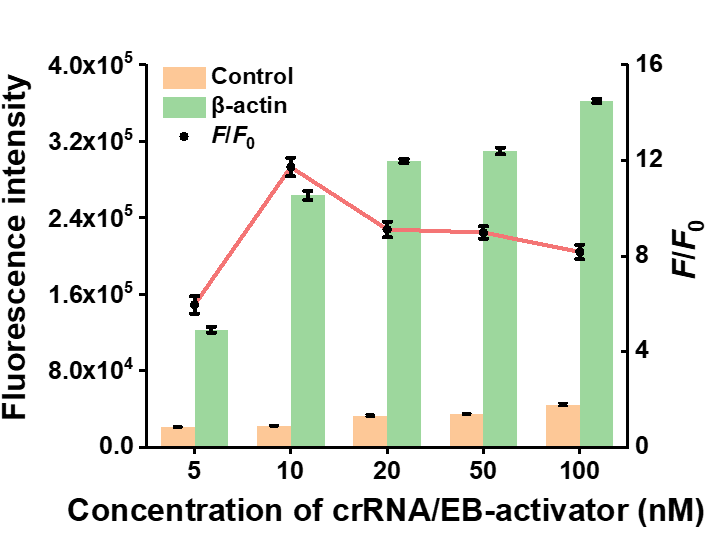


**Figure S6.** Variance of the *F*/*F*_0_ value with different concentrations of crRNA/EB-activator. Data are presented as mean ± SD (*n* = 3).

**Optimization of the concentration of Cas12a**

We further explored the effect of Cas12a upon the assay performance. As shown in Figure S7, the *F*/*F*_0_ value enhances with the increasing concentration of Cas12a from 10 to 50 nM, follow by the decrease beyond the concentration of 50 nM. Thus, 50 nM Cas12a is used in the subsequent researches.


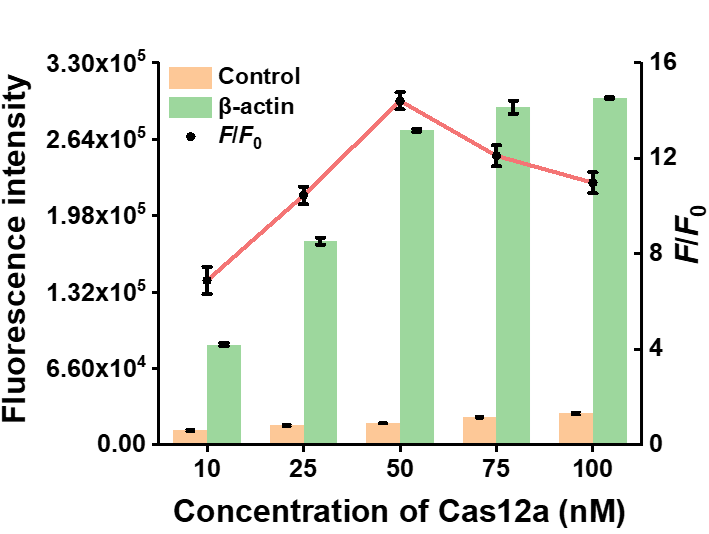


**Figure S7.** Variance of the *F*/*F*_0_ value with different concentrations of Cas12a. Data are presented as mean ± SD (*n* = 3).

**Optimization of the concentration of reporter**

We optimized the concentration of reporter as well. As shown in Figure S8, the *F*/*F*_0_ value improves with the increasing concentration of reporter from 50 to 200 nM, followed by the decrease beyond the concentration of 200 nM. Thus, 200 nM reporter is used in the subsequent researches.


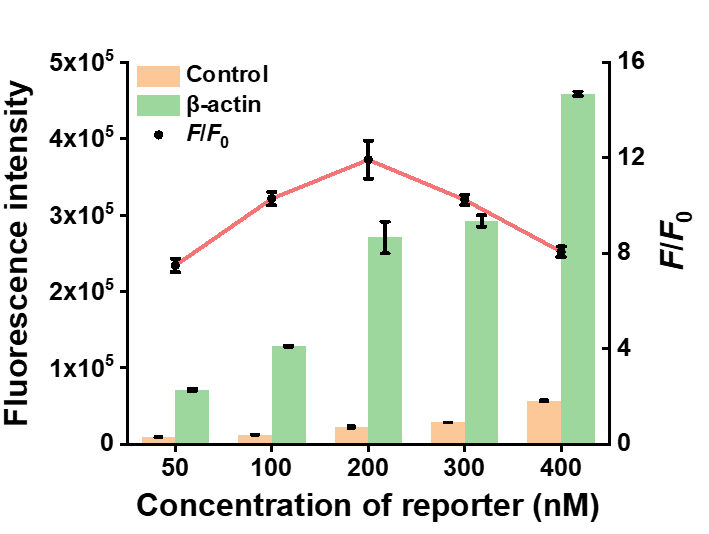


**Figure S8.** Variance of the *F*/*F*_0_ value with different concentrations of reporter. Data are presented as mean ± SD (*n* = 3).

**Optimization of the reaction time**

We optimized the reaction time as well (Figure S9). The *F*/*F*_0_ value enhances with the reaction time from 40 to 60 min, and reaches a plateau at 60 min. Thus, reaction time of 60 min is used in the subsequent researches.


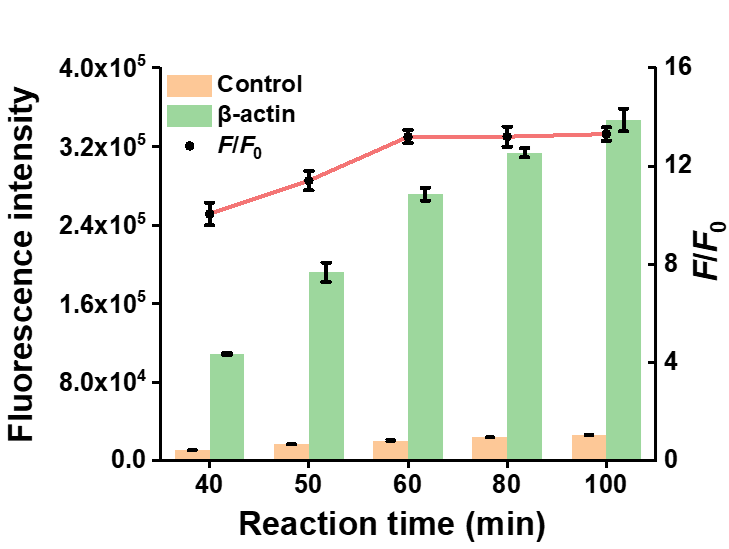


**Figure S9.** Variance of the *F*/*F*_0_ value with reaction time. Data are presented as mean ± SD (*n* = 3).

**Fluorescence emission spectrum of Cas12a@hMNS nanoprobe**


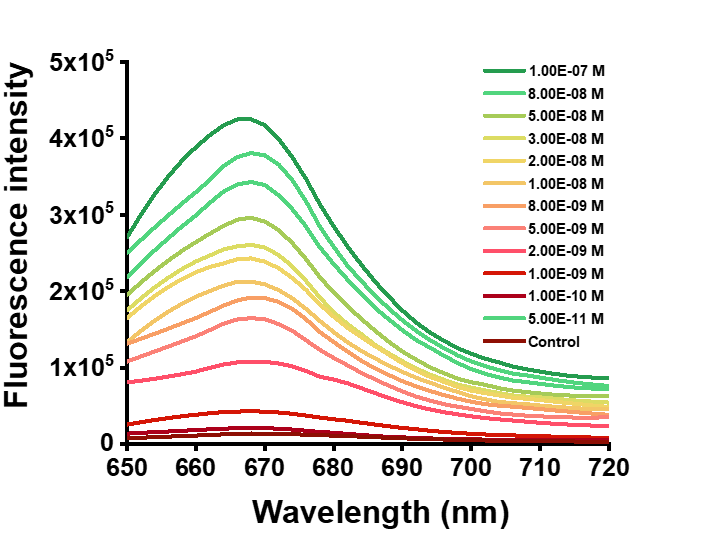


**Figure S10.** Fluorescence emission spectrum of Cas12a@hMNS nanoprobe in response to different concentrations of β-actin mRNA.

**Fluorescence emission spectrum of free CRISPR/Cas12a system**


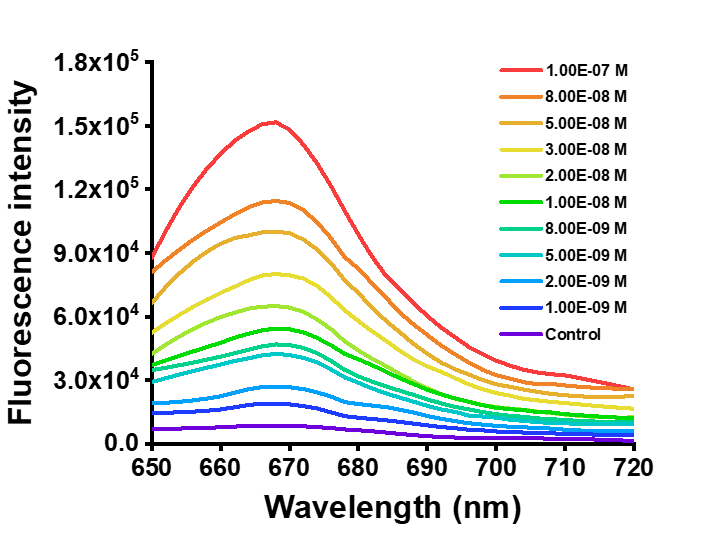


**Figure S11.** Fluorescence emission spectrum of free CRISPR/Cas12a system in response to different concentrations of β-actin mRNA.

**Cytotoxicity assay**


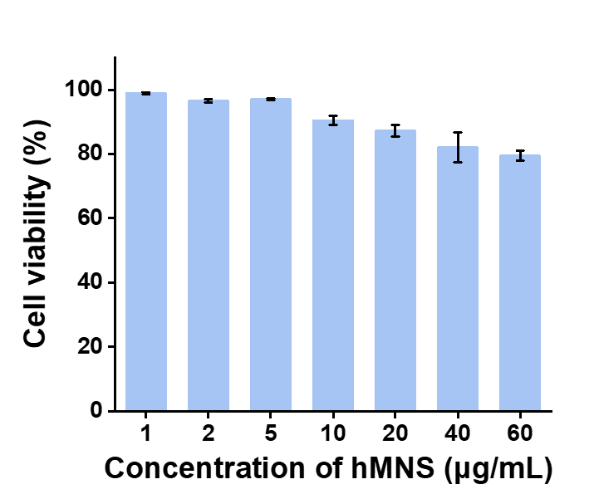


**Figure S12.** Viabilities of MCF-7 cells treated with different concentrations of Cas12a@hMNS nanoprobe for 24 h. Data are presented as mean ± SD (*n* = 3).

**Colocalization assay**


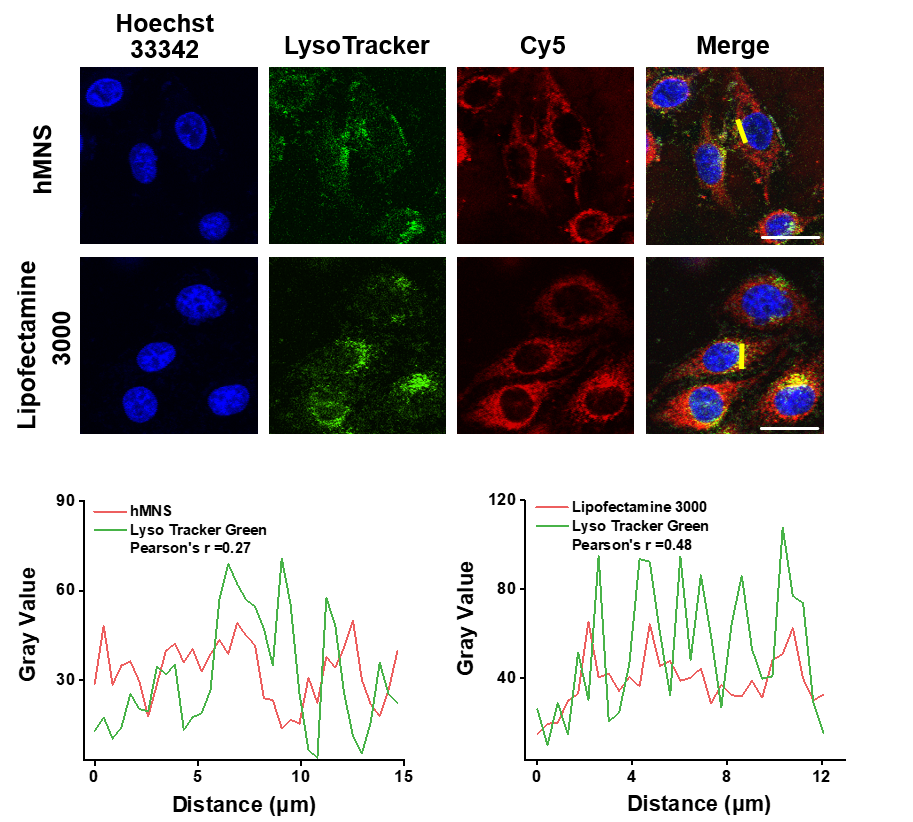


**Figure S13.** Colocalization of lysosome and Cas12a@hMNS or Lipofectamine 3000 in MCF-7 cells. Scale bar: 25 μm.

**References**

[1] H. M. Chen, J. H. He, C. B. Zhang, H. He, Self-assembly of novel mesoporous manganese oxide nanostructures and their application in oxidative decomposition of formaldehyde. *J. Phys. Chem. C* **2007**, *111*, 18033-18038.

[2] J. Wei, H. M. Wang, Q. Wu, X. Gong, K. Ma, X. Q. Liu, F. Wang, A smart, autocatalytic, DNAzyme biocircuit for in vivo, amplified, MicroRNA imaging. *Angew. Chem. Int. Edit.* **2020**, *59*, 5965-5971.
